# Finely Tailored Conjugated Small Molecular Nanoparticles for Near-Infrared Biomedical Applications

**DOI:** 10.34133/research.0534

**Published:** 2025-01-10

**Authors:** Xiaozhen Li, Ruohan Zhang, Yanlong Yang, Wei Huang

**Affiliations:** ^1^ Frontiers Science Center for Flexible Electronics (FSCFE) & Institute of Flexible Electronics (IFE), Northwestern Polytechnical University, Xi’an 710072, P. R. China.; ^2^ Key Laboratory of Flexible Electronics (KLOFE) and Institute of Advanced Materials (IAM), Nanjing Tech University (Nanjing Tech), Nanjing 211816, P. R. China.; ^3^ State Key Laboratory of Organic Electronics and Information Displays and Jiangsu Key Laboratory of Biosensors, Institute of Advanced Materials (IAM), Nanjing University of Posts and Telecommunications, Nanjing 210023, P. R. China.

## Abstract

Near-infrared (NIR) phototheranostics (PTs) show higher tissue penetration depth, signal-to-noise ratio, and better biosafety than PTs in the ultraviolet and visible regions. However, their further advancement is severely hindered by poor performances and short-wavelength absorptions/emissions of PT agents. Among reported PT agents, conjugated small molecular nanoparticles (CSMNs) prepared from D-A-typed photoactive conjugated small molecules (CSMs) have greatly mediated this deadlock by their high photostability, distinct chemical structure, tunable absorption, intrinsic multifunctionality, and favorable biocompatibility, which endows CSMNs with more possibilities in biological applications. This review aims to introduce the recent progress of CSMNs for NIR imaging, therapy, and synergistic PTs with a comprehensive summary of their molecular structures, structure types, and optical properties. Moreover, the working principles of CSMNs are illustrated from photophysical and photochemical mechanisms and light–tissue interactions. In addition, molecular engineering and nanomodulation approaches of CSMs are discussed, with an emphasis on strategies for improving performances and extending absorption and emission wavelengths to the NIR range. Furthermore, the in vivo investigation of CSMNs is illustrated with solid examples from imaging in different scenarios, therapy in 2 modes, and synergistic PTs in combinational functionalities. This review concludes with a brief conclusion, current challenges, and future outlook of CSMNs.

## Introduction

**Fig. 1.**
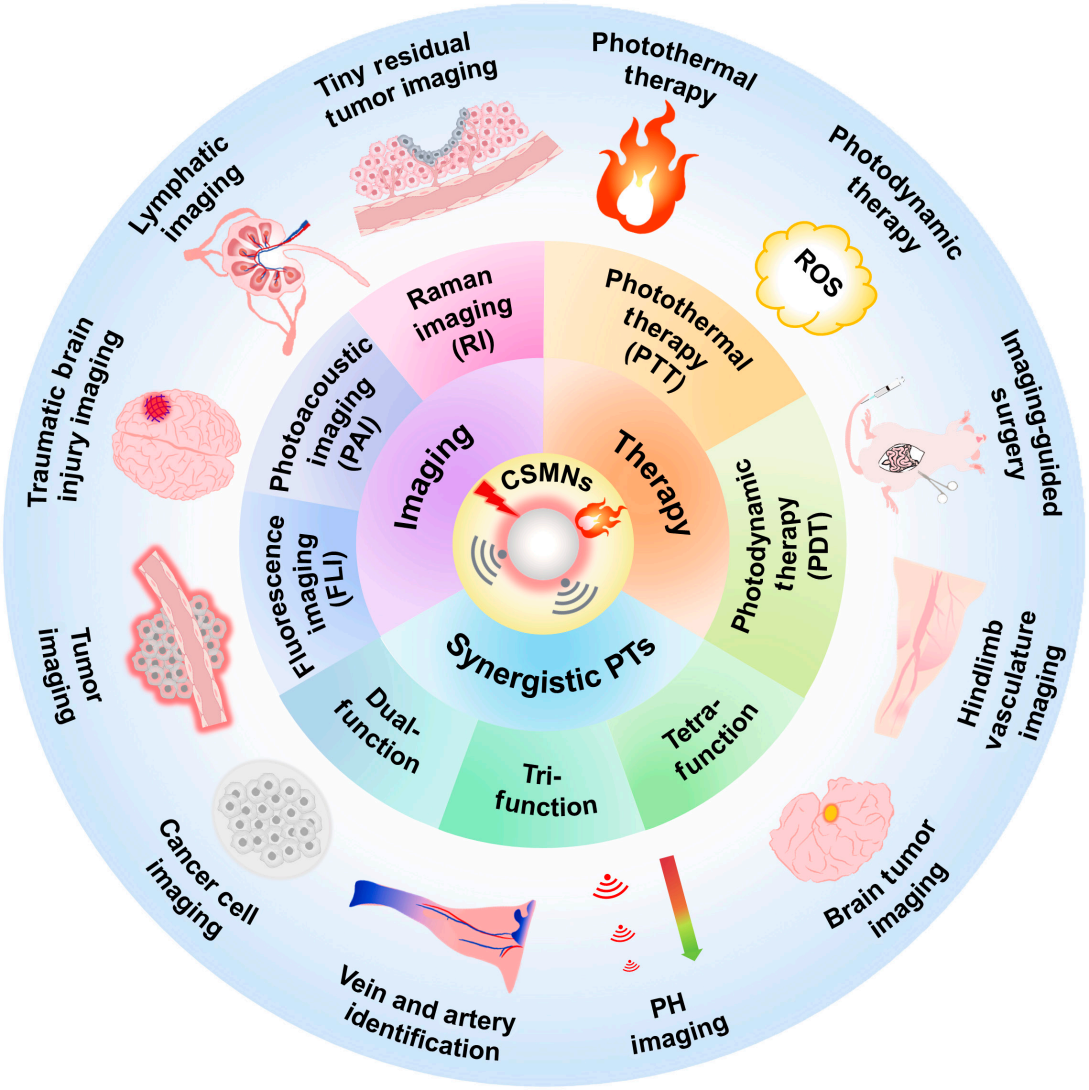
CSMNs for photo-diagnostic technologies (FLI, PAI, and RI) and phototherapies (PTT and PDT).

Near-infrared (NIR) phototheranostics (PTs) have attracted considerable attention from researchers due to their timely monitoring of PT agents and precision therapy of disease in the NIR region [1–12]. Notably, NIR PTs have clarified their superiority over PTs in the ultraviolet (UV) and visible regions regarding tissue penetration depth, signal-to-noise ratio (SNR), and biosafety [4,13–16]. Three photo-diagnostic technologies are generally engaged, including fluorescence imaging (FLI), photoacoustic imaging (PAI), and Raman imaging (RI). NIR FLI can obtain a whole-body image, pinpointing the tumor location [17–23]. PAI utilizes NIR light to stimulate ultrasonic vibration, affording deep penetration and high temporal–spatial resolution [24–30]. RI is featured with a cell-silent region (1,800 to 2,800 cm^−1^), enabling zero-background imaging [31–33]. Phototherapy exploits photoenergy to produce hyperthermia and toxic reactive oxygen species (ROS) for photothermal therapy (PTT) and photodynamic therapy (PDT), respectively [1,34–44]. PDT and PTT have become quite popular owing to their attractive merits, such as noninvasiveness, minimal side effects, and high specificity [45–56]. Freely combining 3 photo-diagnostic technologies (FLI, PAI, and RI) and 2 photo-therapeutic modes (PTT and PDT) endows NIR PTs with more possibilities (Fig. 1) [57–60]. However, further advancement of NIR PTs critically lies in performance optimization and absorption/emission wavelength extension (over 650 nm) of PT agents.

Until now, 2 main categories of NIR PT agents, including inorganic and organic materials, have been reported [9,61–64]. Inorganic nanomaterials show excellent performance in NIR absorbance. However, they are always confronted with long-term toxicity concerns induced by the potential leakage of toxic heavy metal ions and minimal biodegradability, which limits their further clinical translations [65]. For instance, carbon nanotubes were added to the SIN (Substitute it Now) list as the first nanomaterials in 2019 [66]. In contrast, organic nanomaterials, including cyanine dyes, D-A-typed conjugated small molecular nanoparticles (CSMNs), and semiconducting polymeric nanoparticles (SPNs), are more preferred in biological applications because of their good biocompatibility, favorable optical properties, low dark toxicity, and tunable optical properties [48,67–70]. Among them, CSMNs possess higher photostability and tumor accumulation than cyanine dyes [such as Food and Drug Administration-approved indocyanine green (ICG)] [65,71] and more precise molecular structures and higher reliability than SPNs; thus, they have recently emerged as one attractive and promising candidate. The CSMNs are prepared with D-A-typed photoactive CSMs via a nanoprecipitation method or chemical modifications by hydrophilic molecules [such as polyethylene glycol (PEG), affibody, and peptide]. It is worth mentioning that the single photomolecule in CSMNs can achieve more than 1 to 4 functionalities, reflecting the intrinsic multifunctionality of CSMs in CSMNs [72,73]. Such advantages remove the concerns about higher cost and quality control induced by complicated fabrication and compositions.

**Fig. 2.**
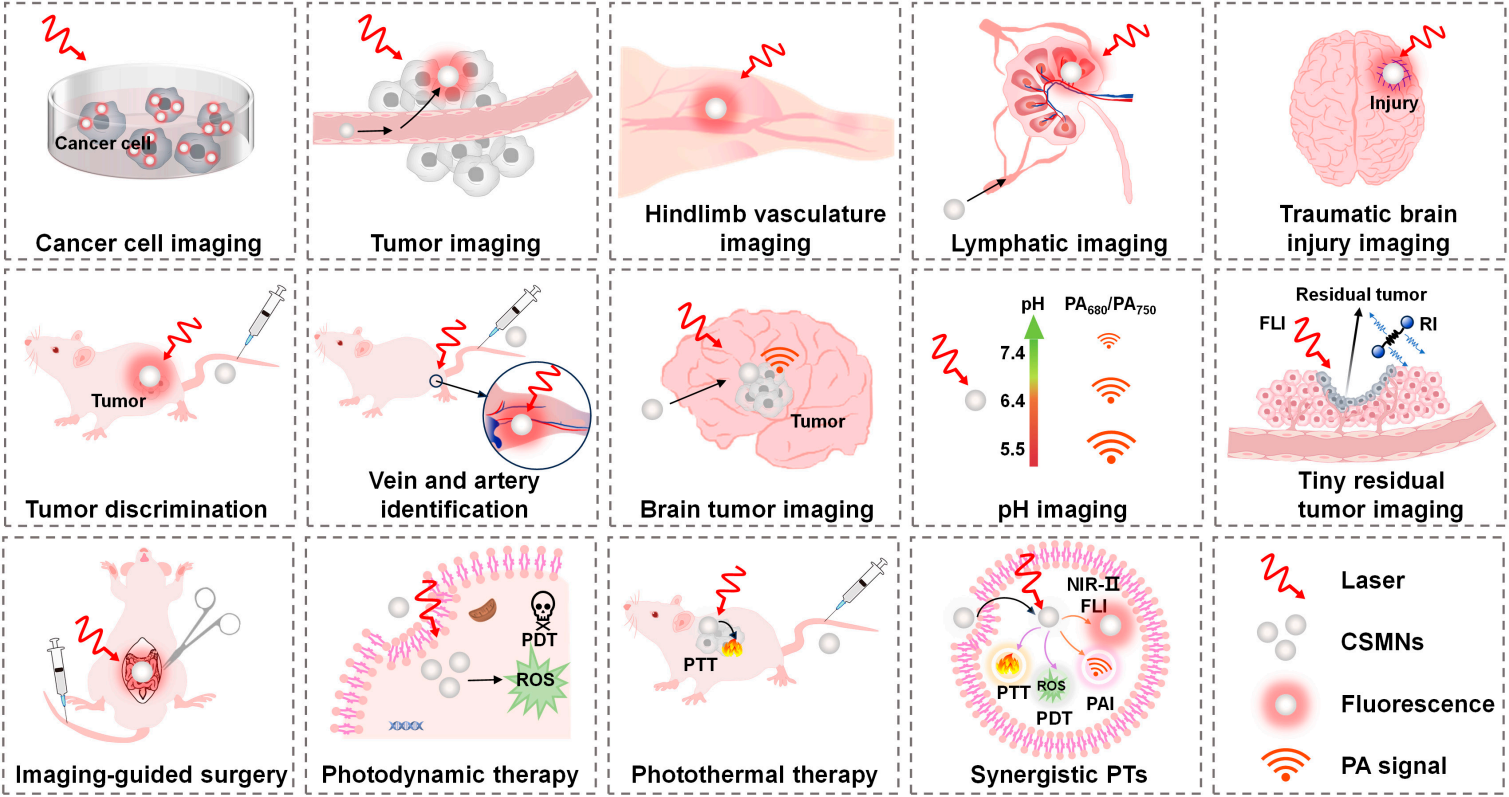
CSMNs for NIR PTs.

Moreover, the molecular structures of CSMs are well defined, while their absorptions can be conveniently adjusted to the NIR region via rational structural design with high repeatability and reproducibility. By these attracting merits, the CSMNs have been applied in the detection of murine hepatoma H22 tumors and cell imaging [74], imaging of cytoplasm of cancer, location of mice bearing breast tumor [75], lymphatic imaging, brain tumor imaging, early-stage detection of head and neck cancers [71], hindlimb vasculature imaging, in-depth lymph node imaging [76], tumor blood vessels and targeted cancer imaging [77], imaging of traumatic brain injury [78], tumor discrimination [79], vein and artery identification [80], imaging of sentinel lymph nodes (SLNs) [81], deep brain tumor imaging [82], in vivo pH imaging [83], identification of tiny residual tumors and portrayal of the margin of tumors, guidance of surgery [84], PTT and PDT against tumor [65,85–89], and imaging (PAI, FLI, RI, or synergistic imaging)-guided tumor therapy (PTT, PDT, or combinational therapy) (Fig. 2) [72,84,90–98]. Therefore, a comprehensive and systematic review focusing on the progress of CSMNs is highly required to provide insights into its applications in both fundamental scientific research and clinical practice.

**Fig.3.**
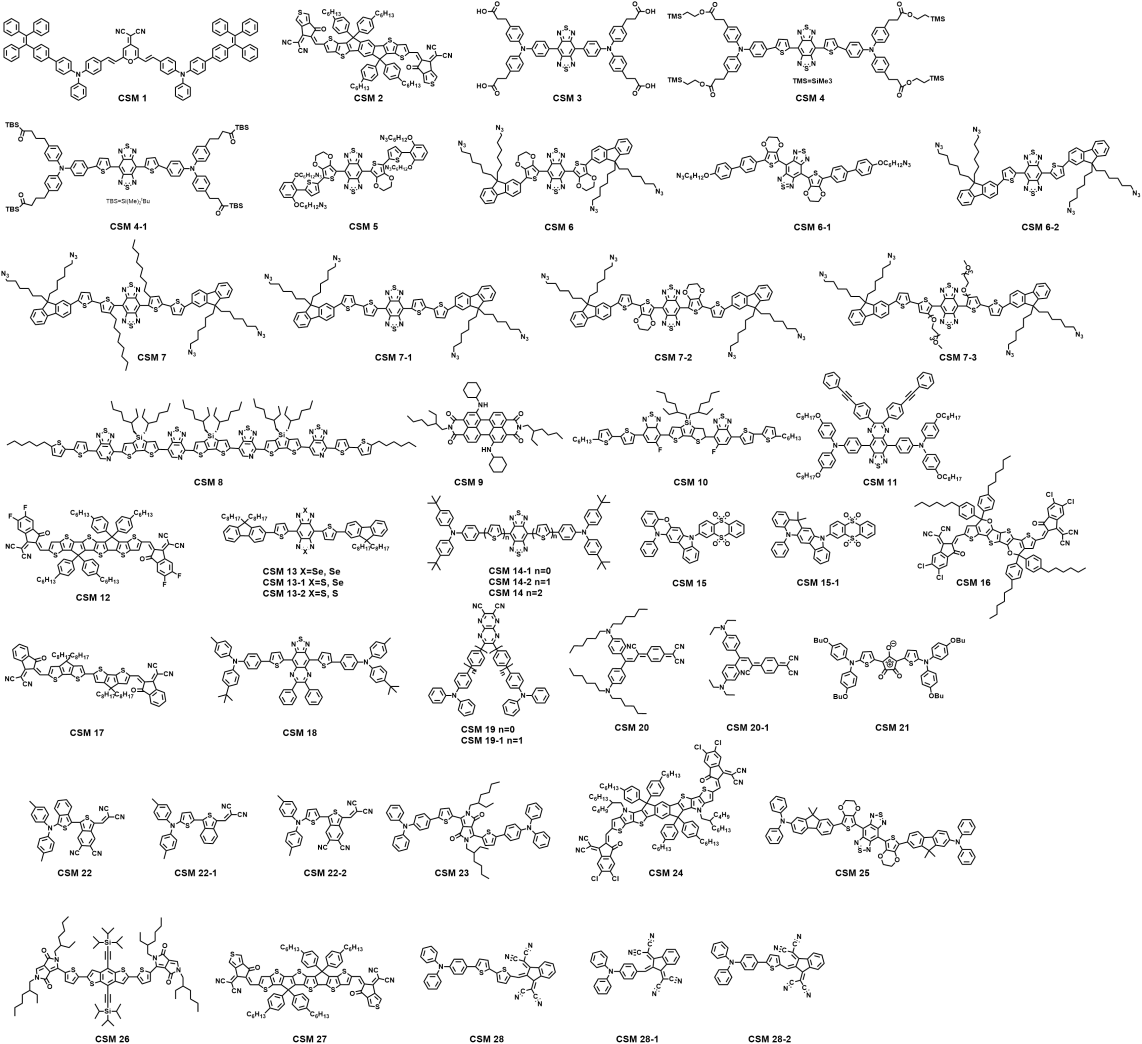
Chemical structures of CSMs for NIR PTs.

**Fig. 4.**
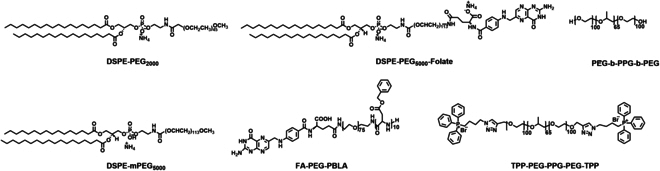
Chemical structures of amphiphilic molecules for encapsulating CSMs.

**Fig. 5.**
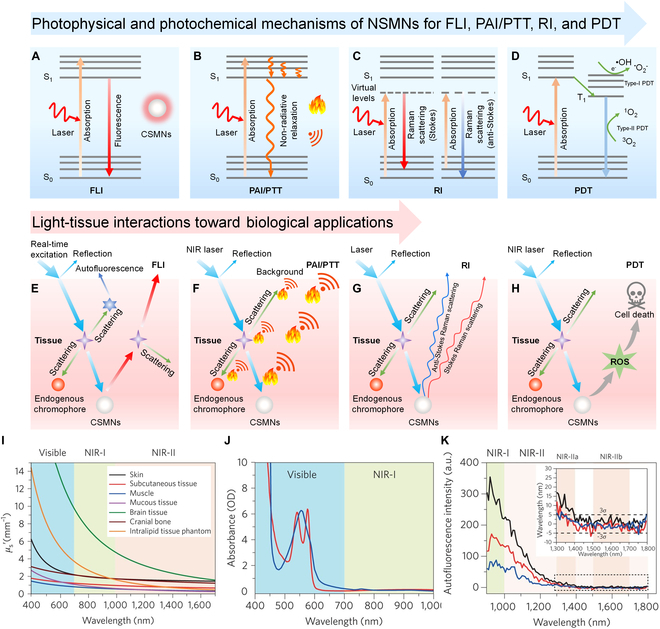
Working principles of CSMNs. Photophysical mechanisms of CSMNs for (A) FLI, (B) PAI/PTT, (C) RI, and (D) PDT. Light–tissue interactions toward biological applications, including (E) FLI, (F) PAI/PTT, (G) RI, and (H) PDT. (I) Scattering coefficients of different biological tissues and tissue phantom as a function of wavelength located within 400 to 1,700 nm. (J) Absorption spectra of deoxyhemoglobin (blue) and oxyhemoglobin (red) in human blood through a 1-mm-long path. (K) Autofluorescence spectra of ex vivo mouse liver (black), spleen (red), and heart tissue (blue) upon exposure to 808-nm laser excitation. Reproduced with permission [100]. Copyright 2017, Springer Nature.

This review covers the recent development of CSMNs for NIR PTs (Fig 1). Their working principles are highlighted in terms of photophysical and photochemical mechanisms of CSMNs for imaging and therapy as well as light–tissue interactions toward biological applications (Fig. 5A to H). Chemical structures of CSMs and amphiphilic polymers for encapsulating CSMs are shown in Figs.3 and 4, respectively. Structural types, optical properties, and applications of currently reported PT agents are summarized comprehensively in Tables 1 to 3. Moreover, molecular and nanoengineering strategies are discussed, with emphasis on improving performances and red-shifting absorption and emission spectra of CSMNs. Then, the biological applications of CSMNs are described from 3 aspects: (a) NIR imaging, including FLI, PAI, and RI in different scenarios; (b) NIR therapy, including PTT and PDT; and (c) synergistic NIR PTs, including dual-function, tri-function, and tetra-function CSMN-mediated NIR PTs. At last, a brief conclusion is given with a discussion of current challenges and possible outlook of CSMNs in the NIR PT field.

**Table 1.** PT agents for NIR imaging

PT agents	Structure type	Encapsulation matrix	λ_ab_ (nm)	λ_ex_ (nm)	λ_em_ (nm)	Φ_f_ (%)	PT mode	Application	References
CSMN 1	D-A-D	DSPE-PEG_2000_ and DSPE-PEG_5000_-folate	354/497	488, 800	600–850	13^a^	NIR-I FLI	Detection of murine hepatoma H22 tumors and cell imaging	[74]
CSMN 2	A-D-A	DSPE-PEG_2000_	≈625/695	635	700–850	7.5^a^	NIR-I FLI	Imaging of cytoplasm of cancer and location of mice bearing breast tumor	[75]
CSM 3-PEG	D-A-D	PEG-modified	≈710	808	900–1,300	0.3^a^	NIR-II FLI	Lymphatic imaging; brain tumor imaging	[71]
CSM 3-affibody	D-A-D	Affibody-modified	/	/	/	/	NIR-II FLI	Early-stage detection of head and neck cancers	[71]
CSM 3-SA	D-A-D	Sulfonic acid-modified	738	808	900–1,300	5/11^b^	NIR-II FLI	Hindlimb vasculature imaging; deep lymph node imaging	[76]
CSMN 4	D-A-D	DSPE-mPEG_5000_	≈830	808	900–1,600	/	NIR-II FLI	Imaging of tumor blood vessels	[77]
CSMN 4-RM26	D-A-D	RM26 modified	/	808	900–1,600	0.2^a^	NIR-II FLI	Targeted imaging of prostate cancer	[75]
CSM 5-PEG	D-A-D	PEG-modified	≈1,100	808	900–1,300	0.7^a^	NIR-II FLI	Traumatic brain injury Imaging	[78]
CSM 6-PEG	S-D-A-D-S	PEG-modified	780	808	900–1,300	2.0^a^	NIR-II FLI	Tumor discrimination	[79]
CSM 6-1-PEG	S-D-A-D-S	PEG-modified	741	808	900–1,300	0.4^a^	NIR-II FLI	/	[79]
CSM 6-2-PEG	S-D-A-D-S	PEG-modified	828	808	900–1,300	0.02^a^	NIR-II FLI	/	[79]
CSM 7-PEG	S-D-A-D-S	PEG-modified	733	808	900–1,350	5.3^a^	NIR-II FLI	Ultrafast vasculature imaging; vein and artery identification	[80]
CSM 7-1-PEG	S-D-A-D-S	PEG-modified	895	808	900–1,350	0.1^a^	NIR-II FLI	/	[80]
CSM 7-2-PEG	S-D-A-D-S	PEG-modified	820	808	900–1,350	1.0^a^	NIR-II FLI	/	[80]
CSM 7-3-PEG	S-D-A-D-S	PEG-modified	784	808	900–1,350	1.4^a^	NIR-II FLI	/	[80]
CSMN 8	/	DSPE-PEG_2000_	710	800	/	/	NIR-I PAI	Sentinel lymph node imaging	[81]
CSMN 9	D-π-A	DSPE-mPEG_5000_	700	700	/	/	NIR-I PAI	Deep brain tumor imaging	[82]
CSMN 10	/	PEG-b-PPG-b-PEG	680/750	680/750	/	/	NIR-I PAI	In vivo imaging of pH	[83]
CSMN 11	D-A-D	DSPE-PEG_2000_	895	704/700/532	800–1,100	2.7	NIR-I FLI/PAI/RI	Precise guidance of cancer surgery	[84]

/, not available; Φ_f_, fluorescence quantum yield

^a^ Measured in water.

^b^ Measured in serum proteins.

**Table 2.** PT agents for NIR therapy

PT agents	Structure type	Encapsulation matrix	λ_ab_ (nm)	λ_ex_ (nm)	PCE (%)	Φ (%)	PT mode	Animal model	References
CSMN 12	A-D-A	DSPE-PEG_2000_	≈750/808	808	82	/	NIR-PAI/PTT	4T1 tumor xenograft mice	[65]
CSMN 13	D-π-A-π-D	DSPE-PEG_2000_	1,120	1,064	77	/	NIR-II PAI/PTT	4T1 tumor xenograft mice	[85]
CSMN 13-1	D-π-A-π-D	DSPE-PEG_2000_	975	1,064	73	/	/	/	[85]
CSMN 13-2	D-π-A-π-D	DSPE-PEG_2000_	830	1,064	61	/	/	/	[85]
CSMN 14	D-A-D	PEG-b-PPG-b-PEG	≈860	808/1,064	28.8/31.6	/	NIR I/NIR-II PAI/PTT	HuH-7 tumor-bearing nude mice	[86]
CSMN 14-1	D-A-D	PEG-b-PPG-b-PEG	≈720	/	19.3/25.3	/	/	/	[86]
CSMN 14-2	D-A-D	PEG-b-PPG-b-PEG	≈820	/	24.1/31.5	/	/	/	[86]
CSMN 15	D-A	DSPE-PEG_2000_	300–400	800	/	13.7	FLI/PDT	/	[87]
CSMN 15-1	D-A	DSPE-PEG_2000_	300–400	800	/	7.7	FLI/PDT	/	[87]
CSMN 16	A-D-A	PS-b-PEG	856/1,035	808	/	2.7	NIR-II FLI/ NIR-I PDT	4T1 tumor-bearing mice	[88]
CSMN 17	A-D-A	FA-PEG-PBLA	750/825	808	36.5	18.6	PDT/PTT	SK-OV-3 tumor-bearing mice	[89]

Φ, ^1^O_2_ quantum yield

**Table 3.** PT agents for synergistic NIR PTs

Categories	PT agent	Structure type	Encapsulation matrix	λ_ab_ (nm)	λ_ex_ (nm)	λ_em_ (nm)	PCE (%)	Φ (%)	Φ_f_ (%)	PT mode	Animal model	References
Dual-function CSMNs	CSMN 18	A-D-A	DSPE-PEG_2000_	≈780	780/808	/	82%	/	/	PAI/PTT	4T1 tumor-bearing nude mice	[90]
	CSMN 19	D-A	DSPE-PEG_2000_	610	660	800–1,100	52	/	/	PAI/PTT	4T1 tumor-bearing nude mice	[91]
	CSMN 19-1	D-A	DSPE-PEG_2000_	610	660	/	59	/	/	PAI/PTT	4T1 tumor-bearing nude mice	[91]
	CSMN 20	D-A	PEGbPPGbPEG	705/836/ 952	/	/	75	/	/	PAI/PTT	4T1 tumor-bearing nude mice	[92]
	CSMN 20-1	D-A	PEGbPPGbPEG	/	/	/	/	/	/	/	/	[92]
	CSMN 21	/	DSPE-PEG_2000_	790	808	/	68	/	/	PAI/PTT	4T1 tumor-bearing nude mice	[93]
Tri-function CSMNs	CSMN 22	D-π-A	DSPE-PEG_2000_	843	808	/	58.3	1.98	/	PAI/PDT/PTT	A549 tumor-bearing nude mice	[94]
	CSMN 22-1	D-π-A	DSPE-PEG_2000_	≈645	/	/	/	/	/	/	/	[94]
	CSMN 22-2	D-π-A	DSPE-PEG_2000_	≈700	/	/	/	2.4	/	/	/	[94]
	CSMN 23	D-A-D	/	660	660	/	34.5	33.6	/	PAI/PDT/PTT	HCT-116 tumor-bearing nude mice	[95]
	CSM 24	A-D-A	TPP-PEG-PPG-PEG-TPP	821	808	850–1,100	39.6	2.3	2.2	NIR-II FLI/PDT/PTT	Hela tumor-bearing mouse	[96]
	CSMN 25	DD-A-DD	DSPE-mPEG_5000_	765	808	800–1,200	21.8	/	/	NIR-II FLI/PAI/PTT	4T1 tumor-bearing nude mice	[97]
Tetra-function CSMNs	CSMN 26	A-D-A	DSPE-mPEG_5000_	625/667/750	660	850–1,200	23	49.3	0.52	NIR-II FLI/PAI/PDT/PTT	HeLa tumor-bearing nude mice	[98]
	CSMN 27	A-D-A	DSPE-PEG_2000_	≈700/800	808	900–1,200	52.8	0.61	3.0	NIR-II FLI/PAI/PDT/PTT	Nude mice bearing A549 tumor xenograft	[72]
	CSMN 28	D-π-π-A	DSPE-PEG_2000_	≈580/630	660	800–1,200	46	/		NIR-II FLI/PAI/PDT/PTT	4T1 tumor-bearing nude mice	[73]
	CSMN 28-1	D-A	DSPE-PEG_2000_	≈580/620	660	800–1,150	/	/		/	/	[73]
	CSMN 28-2	D-π-A	DSPE-PEG_2000_	≈580/630	600	800–1,150	/	/		/	/	[73]

## Working Mechanism

The working mechanisms of CSMNs are illustrated from 2 perspectives. The first is the photophysical and photochemical mechanisms of CSMNs for FLI, PAI/PTT, RI, and PDT. Upon exposure to a laser, the molecules in CSMNs will firstly absorb incident light to be excited from the ground state (S_0_) to the excited singlet state (S_1_). As the Jablonski diagram shows, the excited molecules can dissipate energy via several pathways [99]. If the excited population dissipates their energy from S_1_ to S_0_ via the radiative decay, the molecules can emit fluorescence for FLI (Fig. 5A). If the excited population loses their energy from S_1_ to S_0_ via nonradiative decay, the molecules can release heat for PAI and PTT (Fig. 5B). If the excited population undergoes intersystem crossing (ISC) to triplet state (T_1_), there appear another 3 paths for excited molecules to release energy: (a) phosphorescence from T_1_ to S_0_; (b) energy transfer from T_1_ to surrounding O_2_ to produce ^1^O_2_; and (c) electron transfer from T_1_ to surrounding substrates to generate free radicals, such as O_2_^·−^ and •OH. The produced ^1^O_2_ and free radicals (O_2_^·−^ and •OH) can be used for type I and II PDT, respectively (Fig. 5D). For the Raman effect, the photons excite the molecule from S_0_ to a virtual energy state. When the excited molecules emit photons and return to a rotational or vibrating state different from S_0_, the energy difference between S_0_ and the new state causes the frequency of the emitted photons to differ from the wavelength of the excited light. If the molecules in the final vibrational state have a higher energy than the initial state, the excited photons have a lower frequency to ensure that the total energy of the system is maintained. This change in frequency is called the Stokes shift. If the final vibrational state of the molecule is lower in energy than the initial state, the excited photon frequency is higher, and this frequency change is called the anti-Stokes shift (Fig. 5C).

The second is light–tissue interactions toward biological application [100]. When the excitation light (usually a single light source) impinges on the surface of the objects of interest, a large portion of the incident light is reflected at the air/tissue interface due to the difference in refractive index between air and superficial tissue. The remaining incident light continuously penetrates the tissue and encounters scattering events, induced by the inhomogeneity of refractive exponents of different components of animal tissue (such as water, lipid membrane, and subcellular organelles). Scattering processes further weaken the signal for biological application and add background noise. Empirical measurements of scattering in tissues revealed an inversely proportional relationship between the scattering coefficient and wavelength (Fig. 5I), suggesting reduced scattering for all tissue types (including brain tissue, skin, cranial bone, mucous tissue, subcutaneous tissue, and muscle) at a longer wavelength. Endogenous chromophores such as deoxyhemoglobin and oxyhemoglobin show strong absorption in the <600-nm range (Fig. 5J). Except for UV–visible absorption from endogenous chromophores, water also displays a substantial contribution to the absorption spectrum of biological tissue with absorption maxima at 970, 1,200, 1,450, and beyond 1,800 nm, setting the boundaries of suitable optical windows for deep-tissue imaging. With consideration of the extinction spectra from all major light absorbers in biological tissue, the entire 700- to 1,700-nm spectral range (except a narrow water absorption centered at 1,450 nm) should permit deep-tissue imaging benefiting from low scattering and absorption of both incident and emissive photons. Autofluorescence from vital organs and bodily fluids is the fourth limiting factor for tissue penetration depth, which also decreases with the increase of detection wavelength but appears as a trailing tail to the NIR window and disappears beyond 1,500 nm (Fig. 5K). After the incident light encounters reflection, scattering, endogenous absorption, and autofluorescence, major photons will be absorbed by CSMs in CSMNs to be excited from S_0_ to S_1_ (virtual state for Raman scattering). According to different energy-dissipate pathways, including radiative decay, nonradiative decay, scattering, and ISC of CSMs, they can be used for FLI (Fig. 5E), PAI/PTT (Fig. 5F), RI (Fig. 5G), and PDT (Fig. 5H), respectively.

## Strategies for Regulating the Absorptions of CSMs to the Second NIR Window (NIR-II)

**Fig. 6.**
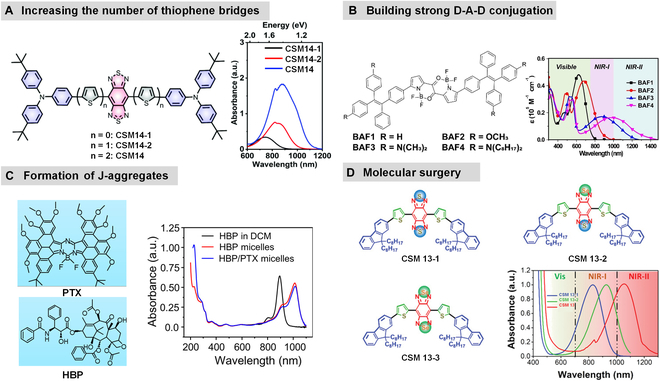
Strategies for regulating the absorption of CSMs to NIR-II. (A) Increasing the number of thiophene bridges. Reproduced with permission [86]. Copyright 2020, Royal Society of Chemistry. (B) Building strong D-A-D conjugation. Reproduced with permission [101]. Copyright 2021, Wiley-VCH. (C) Formation of J-aggregates. Reproduced with permission [102]. Copyright 2020, Elsevier. (D) Molecular surgery. Reproduced with permission [85]. Copyright 2020, Wiley-VCH.

Because of the design difficulty, only a handful of CSMNs were reported for NIR-II PAI. To regulate the absorption of CSMs to NIR-II, several strategies, including increasing the number of thiophene bridges, building strong D-A-D conjugation, formation of J-aggregates, and molecular surgery, have been reported (Fig. 6).

### Increasing the number of thiophene bridges

Ling’s group [86] synthesized 3 D-A-D structured CSMs (CSM 14, CSM 14-1, and CSM 14-2) using triphenylamine (TPA) as the donor and benzo[1,2-c:4,5-c′]bis([1,2,5]thiadiazole) (BBT) as the acceptor, in which the number of thiophene bridges increased sequentially (0 to 2) (Fig. 6A). With the prolonged thiophene bridges, the absorption spectra extended from the first NIR window (NIR-I) to NIR, which might be attributed to the extension of the effective conjugation length of the molecular backbone. Notably, CSM 14-2 exhibited an absorption spectrum ranging from 700 to 1,200 nm, covering both NIR-I and NIR-II. Experimental results confirmed the feasibility and effectiveness of extending the optical absorption of CSMs into the NIR-II region by increasing the number of thiophene bridges in the molecular backbone.

### Building strong D-A-D conjugation

Liu and colleagues [101] integrated a strong electron donor, 1,2-bis(4-*N*,*N*-dioctylaminophenyl)-1,2-diphenylmethane, into a difluoroboron-bridged azacyclic dimeric framework to design a CSM, BAF4, with a strong D-A-D conjugated system (Fig. 6B). Compared with the BAF4 analogs, BAF1 to BAF3 (with altered donor structure), BAF4’s absorption peak red-shifted to 1,000 nm, with a maximum absorption wavelength exceeding 1,400 nm. Theoretical calculations further indicated the smallest energy gap of BAF4 containing strong electron-donating groups, theoretically supporting the design strategy. Thus, increasing the electron-donating ability of the donor to enhance the conjugation coupling of the main chain can significantly boost the NIR-II absorption of D-A-D structured small molecules.

### Formation of J-aggregates

Feng’s team [102] constructed an HBP/PTX nanoaggregate by self-assembling a cyclically fused azaBODIPY (referred to as HBP) with the hydrophobic paclitaxel (PTX) [102] (Fig. 6C). UV–visible–NIR absorption spectra showed that the absorption peak of HBP red-shifted from 878 to 1,012 nm, verifying the formation of J-aggregates. Thus, the successful construction of J-aggregates can also achieve a redshift of the absorption peak to NIR-II.

### Molecular surgery

Lee and colleagues [85] designed a D-π-A-π-D CSM (CSM 13-1) using a twisted fluorene as the donor, BBT as the acceptor, and thiophene as the π-electron bridge for intramolecular charge transfer (ICT). Based on this, they performed substitution of the sulfur (S) atom in the BBT unit with selenium (Se) atoms, synthesizing structurally similar molecules CSM 13-2 and CSM 13 with different numbers of Se atoms (Fig. 6D). The absorption peaks of CSM 13-2 and CSM 13 both red-shifted to varying degrees compared to CSM 13-1. CSM 13 with 2 Se atoms showed an absorption peak at 1,060 nm. Via this molecular surgery of single-atom replacement, the absorptions of CSMs could be effectively regulated from NIR-I to NIR-II.

## Imaging

Imaging techniques provide wealthy physiological and pathological information of living subjects beneficial for precise disease diagnosis [100,103]. Among various imaging agents, CSMNs have recently attracted extensive research interest and have been explored as contrast agents in imaging [104]. In this part, the imaging applications of CSMNs, including FLI, PAI, and RI, will be investigated. Recently developed CSMNs for NIR imaging are summarized in Table 1.

### Fluorescence imaging

As an emerging class of fluorescence probes, CSMNs have high fluorescence quantum yields (QYs) and structural reliability. Due to those charming merits, various CSMNs have been designed and explored for NIR FLI.

#### NIR-I FLI

In past decades, CSM-based fluorophores emitted fluorescence mainly in NIR-I (650 to 900 nm).

**Fig. 7.**
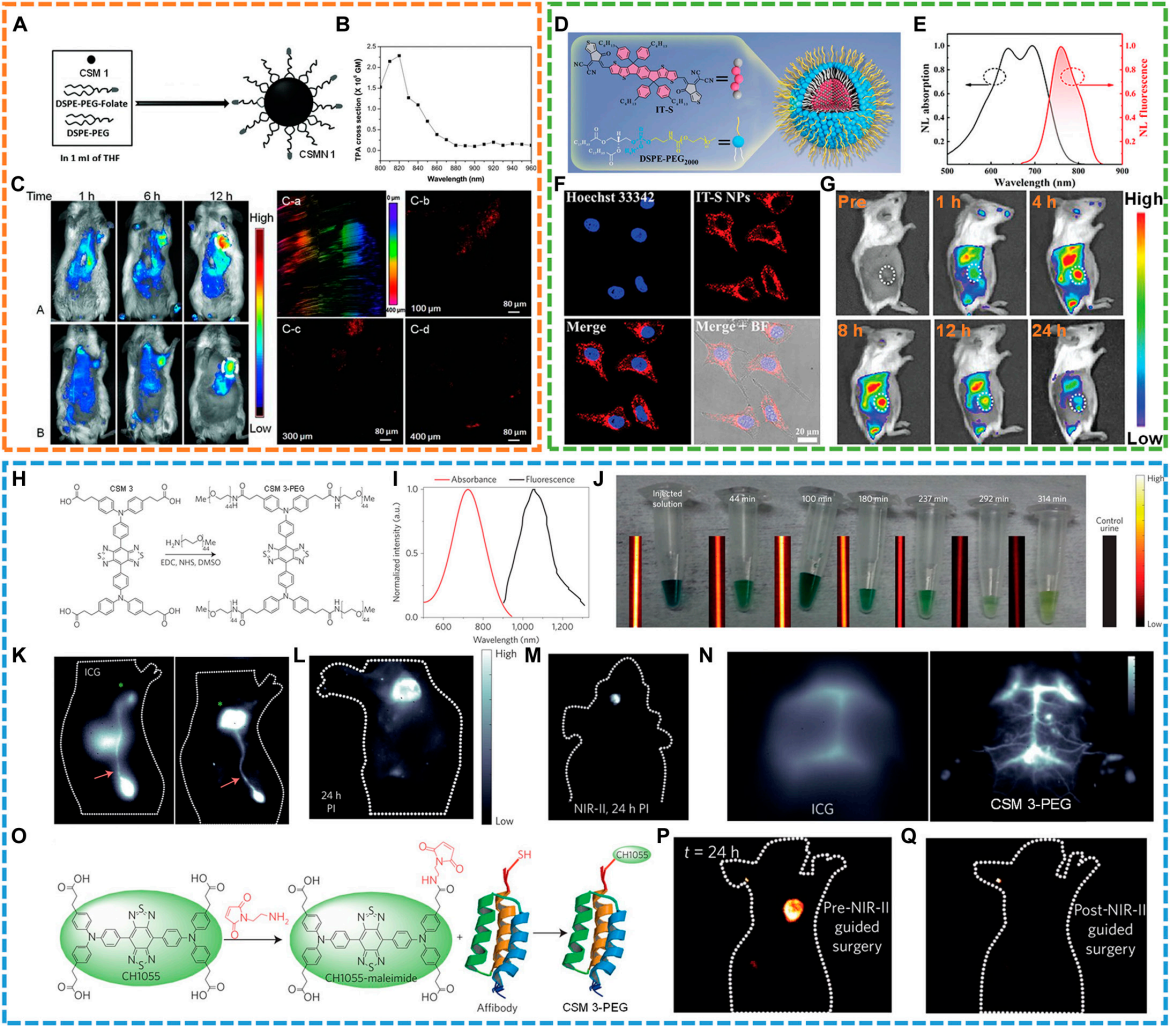
NIR-I and NIR-II FLI. (A) Schematic of CSMN 1 synthesis. (B) Two-photon absorption cross-section measurement. (C) In vivo one-photon FLI (left) and 2-photon imaging of isolated tumor tissues (right). Reproduced with permission [74]. Copyright 2012, Wiley-VCH. (D) Schematic illustration of CSMN 2 preparation. (E) Absorption and emission spectra of CSMN 2. (F) Confocal imaging of cancer cells incubated with CSMN 2. (G) In vivo FLI after injection of CSMN 2. Reproduced with permission [75]. Copyright 2020, Wiley-VCH. (H) Modification of PEG on side chain of CSM 3. (I) Absorbance and emission spectra of CSM 3-PEG. (J) Pictures of injected solution and collected urine sample as well as their corresponding NIR-II fluorescence. (K) In vivo NIR-II fluorescence of tumor, lymph vasculature, and lymph node with ICG or CSM 3-PEG. (L) NIR-II FLI of living mouse at 24 h after injection of CSM 3-PEG. (M) NIR-II FLI for brain tumor. (N) Brain vasculature imaging with ICG or CSM 3-PEG. (O) Modification of affibody on the side chain of CSM 3. NIR-II imaging of tumor (P) before and (Q) after surgery. Reproduced with permission [71]. Copyright 2015, Springer Nature.

Detection of murine hepatoma H22 tumors and cell imaging. In 2012, Liu and colleagues [74] reported a bright fluorophore (CSM 1) with large 2-photon absorption (TPA) cross section and NIR emission for 1-photon and 2-photon imaging (Fig. 7A to C). To endow the molecule with good water solubility and cancer cell-targeting ability, CSM 1 was encapsulated into water-dispersible nanoparticles (CSMN 1, ~50 nm) with both DSPE-PEG_2000_ and DSPE-PEG_5000_-folate (Fig. 7A). One-photon in vivo FLI demonstrated that CSMN 1 could be effectively taken in by murine hepatoma H22 tumors with the assistance of folate-motivated active targeting effect and permeability and retention (EPR) effect. Two-photon cell imaging showed no autofluorescence from cells, and the ex vivo imaging achieved deep tumor imaging (~400 μm) (Fig. 7C). All results indicated the great potential of CSMN 1 for future imaging and diagnostic applications.

Imaging of cytoplasm of cancer and mice bearing breast tumor. More recently, our group presented a conjugated oligomer-based nanotherapeutic (CSMN 2) (Fig. 7D) with a high mass extinction coefficient of 93.5 l g^−1^ cm^−1^ and photoluminescence QY (PLQY) of 7.5% for high-performance cancer theranostics [75]. CSMN 2 showed broad absorption from 550 to 750 nm and a relatively narrow emission spectrum peaking at 760 nm (Fig. 7E). The cytoplasm of the cancer cell was delineated by red fluorescence from CSMN 2 (Fig. 7F), which demonstrated efficient endocytosis of CSMN 2. Moreover, CSMN 2 was applied for in vivo FLI, which exhibited maximum fluorescence signal in tumor location at 8 h after intravenous injection of CSMN 2 to mice bearing breast tumor (Fig. 7G). This in vivo imaging experiment thus guided the following cancer therapy.

#### NIR-II FLI

Compared with NIR-I fluorescence, fluorescence in NIR-II (1,000 to 1,700 nm) possesses deeper penetration depth and higher SNR because of reduced photoscattering and tissue autofluorescence [105–108]. Thus, recently, many efforts have been devoted to developing CSMs with NIR-II emission.

Lymphatic imaging, brain tumor imaging, early-stage detection of head and neck cancers, and imaging-guided removal of SLNs and squamous cell carcinoma. For instance, Dai et al*.* [71] synthesized an advanced small molecule (CSM 3) for NIR-II FLI. Before application, CSM 3 was modified with PEG to ensure its good water solubility and small size (Fig. 7H). CSM 3-PEG showed a small size of 3 nm and bright NIR-II fluorescence (QY: 0.3%) peaking at 1,055 nm (Fig. 7I). Pharmacokinetic experiments indicated that about 90% of CSM 3-PEG could be excreted from renal within 24 h after intravenous injection (Fig. 7J). First, CSM 3-PEG was used for lymphatic imaging. CSM 3-PEG mainly accumulated in the tumor, lymphatic vessel, and nodes, whereas most ICG entered the liver, the lymphatic vessel, and nodes, with only a weak signal in the tumor site (Fig. 7K). Moreover, compared with ICG, CSM 3-PEG with lower QYs showed a 2-fold enhancement in lymph node signal-to-background ratio (SBR). Even after 24-h injection of CSM 3-PEG, the tumor and SLN could be concurrently distinguished (Fig. 7L), which enabled selective removal of SLNs via surgical resection to prevent tumor metastasis. Second, CSM 3-PEG was applied for brain tumor imaging, in which brain tumors and vasculatures were visualized in high clarity by NIR-II imaging with CSM 3-PEG. By contrast, relatively low imaging quality was obtained by NIR-I imaging with ICG (Fig. 7M and N). At last, CSM 3 was modified with a small protein anti-epidermal growth factor receptor (EGFR) affibody (CSM 3-affibody) for early-stage detection of head and neck cancers in which EGFR was overexpressed (Fig. 7O). As expected, CSM 3-affibody showed great affinity to squamous cell carcinoma (SAS)-overexpressing EGFR in vitro imaging. The tumor-to-normal tissue ratio could reach ~15 within 6-h injection of CSM 3-affibody, 5 times higher than previously reported values. In vivo imaging further proved the specific targeting ability of CSM 3-affibody to SAS. Considering that CSM 3-affibody-mediated NIR-II imaging could clearly distinguish tumor and normal tissue (Fig. 7P), surgery was performed to remove the tumor site. After surgery, no NIR-II fluorescence signal could be detected from the mouse (Fig. 7Q), indicating the complete removal of SAS, which highlighted the superiority of CSM-mediated NIR-II FLI.

**Fig. 8.**
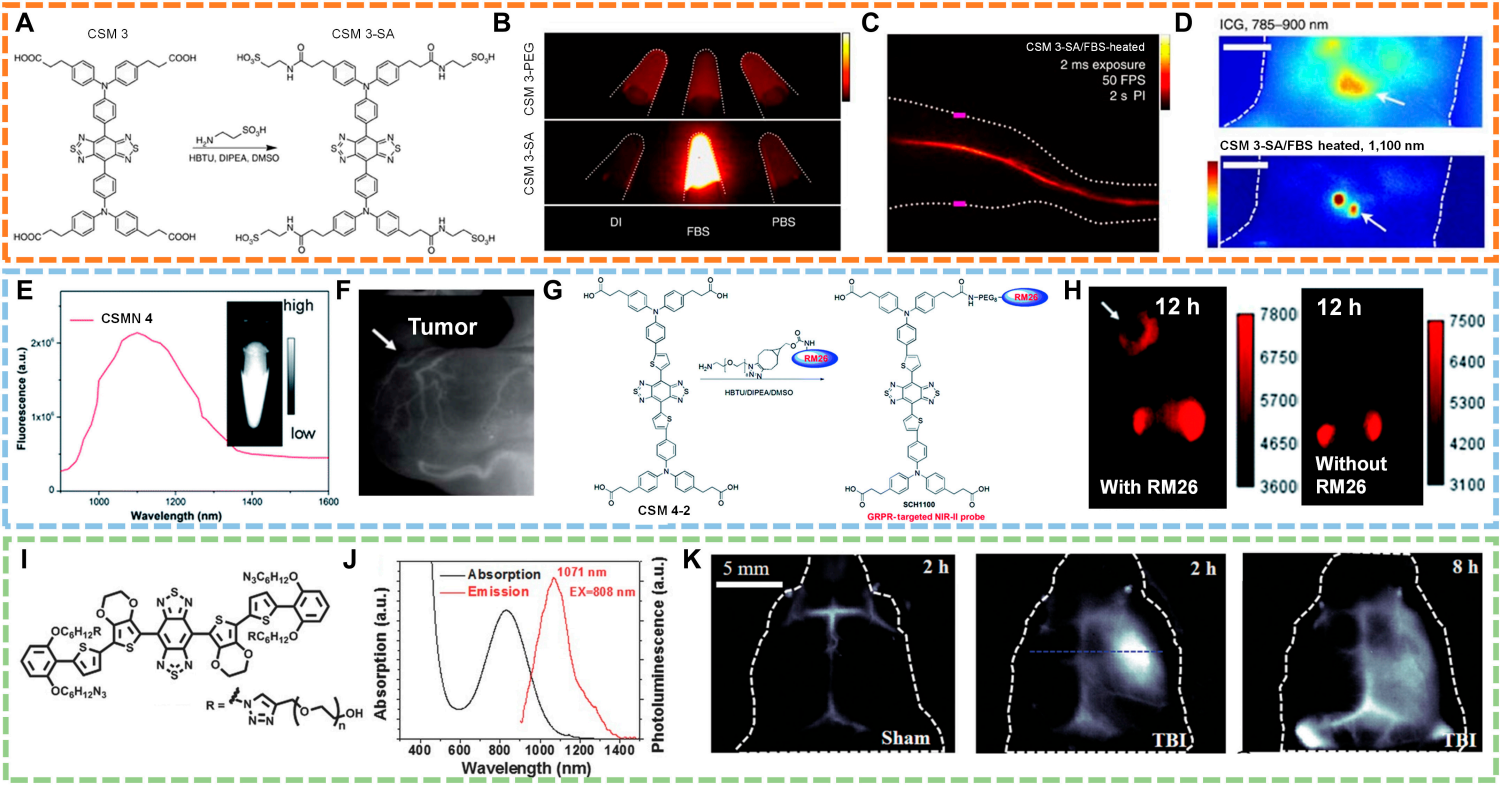
NIR-II FLI. (A) Modification of sulfonic acids on the side chain of CSM 3. (B) In vitro NIR-II FLI of CSM 3-SA in a different medium [including deionized water (DI) water, fetal bovine serum (FBS), and PBS]. (C) NIR-II FLI of hindlimb vasculature at 2-s post-injection of CSM 3-SA/FBS (heated). (D) NIR-I/II FLI of lumbar lymph nodes after injection of ICG and CSM 3-SA/FBS (heated) into both footpads at an exposure time of 300 and 400 ms, respectively. Reproduced with permission [76]. Copyright 2017, Springer Nature. (E) Fluorescence spectrum of CSMN 4. The inset is a NIR-II image. (F) NIR-II imaging of tumor blood vessels at 4 h after intravenous injection of CSMN 4 under 808-nm excitation. (G) Linkage of CSM 4-2 with NH_2_-PEG8-RM26 peptide to prepare a gastrin-releasing peptide receptor (GRPR)-targeted NIR-II probe (SCH1100). (H) NIR-II FLI of PC3 tumor-bearing mice after intravenous injection of SCH1100 with or without RM26 modification under 808-nm irradiation. Reproduced with permission [77]. Copyright 2016, Royal Society of Chemistry. (I) Molecular structure of CSM 5-PEG. (J) Absorption and emission spectrum of CSM 5-PEG. (K) NIR-II FLI of a sham mouse brain and the brain of mouse with traumatic brain injury at 2 or 8 h after injection of CSM5-PEG. Reproduced with permission [78]. Copyright 2016, Wiley-VCH.

Imaging of hindlimb vasculature and lumbar lymph nodes. Based on this work, Chen’s group [76] converted the carboxylic of CSM 3 to sulfonic acids (CSM 3-SA) (Fig. 8A) and found that CSM 3-SA in serum proteins (QY: 5%) displayed a 110-fold fluorescence increment than that in phosphate-buffered saline (PBS) (Fig. 8B), which was due to the formation of supramolecular assemblies between CSM 3-SA and serum proteins. With temperature increase, the fluorescence became much brighter with QY of 11% potentially derived from optimized interaction between CSM 3-SA and proteins. High-fidelity hindlimb vasculature imaging in the NIR-II window at the fastest frame rate so far was achieved with the CSM 3-SA/protein complex (Fig. 8C). Lumbar lymph node imaging further revealed the superiority of NIR-II imaging over NIR-I in deciphering deep anatomical information (Fig. 8D). This work brought hopes for developing NIR-II fluorophores with ultrahigh QYs.

Imaging of tumor blood vessels and targeted cancer imaging. Another new type of NIR-II fluorophore (CSM 4) with a different core structure from CSM 3 was designed first by Cheng’s group [77], in which a thiophen spacer was introduced to the molecular backbone leading to an emission wavelength extension to ~1,400 nm (Fig. 8E). Based on CSM 4, 2 biocompatible probes (CSMN 4 and CSM-RM26) were constructed for NIR-II tumor blood vessels and targeted cancer FLI, respectively (Fig. 8F to H). This work expanded the diversity of NIR-II fluorophores adapting for various applications of NIR-II imaging technique.

Imaging of traumatic brain injury. To improve QYs of CSMs, Dai’s group [78] utilized thiophene-based units as the donor and benzo[1,2-c:4,5-c′]bis([1,2,5]thiadiazole) (BBTD) as the acceptor to construct a new NIR-II fluorophore (CSM 5) with narrow-bandgap “D-A-D” structure, in which bulky 3,4-ethylenedioxy thiophene (EDOT) was introduced to avoid conjugated backbone interaction for further improving of the QYs. Moreover, CSM 5 was modified with PEG chains (CSM 5-PEG) to gain water solubility (Fig. 8I). As expected, CSM 5-PEG showed an emission peak at 1,071 nm and enhanced QYs of 0.70% in an aqueous solution (Fig. 8J). With these properties, CSM 5-PEG was applied for imaging of traumatic brain injury (Fig. 8K).

**Fig. 9.**
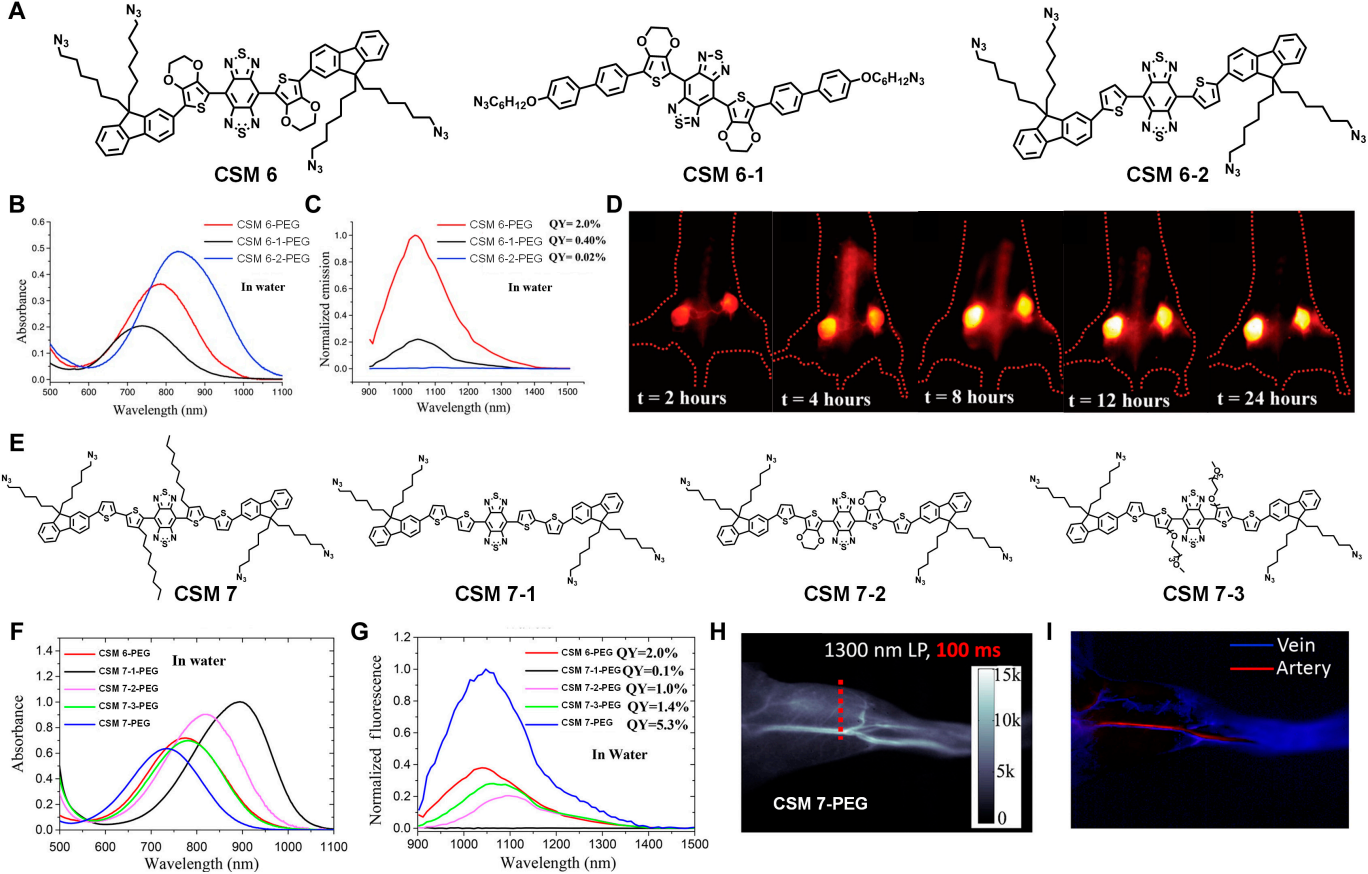
NIR-II FLI. (A) Molecular structures of CSM 6, CSM 6-1, and CSM 6-2. (B) Absorbance and (C) emission spectra of CSM 6-PEG, CSM 6-1-PEG, and CSM 6-2-PEG. (D) NIR-II FLI of 4T1 tumor-bearing mice at different time points (2, 4, 8, 12, and 24 h) after intravenous injection of CSM 6-PEG. Reproduced with permission [79]. Copyright 2016, Wiley-VCH. (E) Molecular structures of CSM 7, CSM 7-1, CSM 7-2, and CSM 7-3. (F) Absorption and (G) emission spectra of CSM 6-PEG, CSM 7-1-PEG, CSM 7-2-PEG, CSM 7-3-PEG, and CSM 7-PEG. (H) Ultrafast NIR-II FLI of vasculature by using CSM 7-PEG. (I) NIR-II FLI of vein and artery by using CSM 7-PEG. Reproduced with permission [80]. Copyright 2018, American Chemical Society.

Tumor discrimination. Although CSMs have obtained initial success in NIR-II imaging, their QYs remain to be improved. Another work from Dai et al*.* [79] reported a shielding unit (S)–donor (D)–acceptor (A)–donor (D)–shielding unit (S) (S-D-A-D-S) structure to optimize the fluorescence performance for biological imaging. Specifically, BBTD was employed as the acceptor, EDOT/thiophen as the donor, and dialkyl fluorene/dibenzene as the shielding unit. According to this design guideline, 3 fluorophores (CSM 6, CSM 6-1, and CSM 6-2) were designed (Fig. 9A). The geometric, electronic, and optical properties of 3 molecular fluorophores are theoretically examined using density functional theory (DFT) and time-dependent density functional theory (TDDFT). Via PEGylation, water-soluble fluorophores (CSM 6-PEG, CSM 6-1-PEG, and CSM 6-2-PEG) showed red-shifted absorption in water, with peak absorption at 780, 828, and 741 nm (Fig. 9B), respectively. The QYs of CSM 6-PEG, CSM 6-1-PEG, and CSM 6-2-PEG were measured to be 2.0%, 0.02%, and 0.40% (Fig. 9C), respectively. The results confirmed that both the EDOT as the donor and dialkyl fluorene as the shielding unit accounted for the highest QY of CSM 6-PEG because they can protect the conjugated backbone from intermolecular and intramolecular interactions. CSM 6-PEG was thus applied for tumor discrimination (Fig. 9D).

Vein and artery identification. They also adopted a donor engineering strategy for further improving QYs, from which the relationship between the donor structure and QYs was illuminated [80]. The fluorophores all contain benzo[1,2-c:4,5-c′]bis[1,2,5]thiadiazole (BBTD) as acceptor, fluorene as shielding unit, and thiophen as the second donor; the only variable was the first donor connected to the acceptor (Fig. 9E). Results showed longer absorption wavelength due to the added thiophene for extended conjugation and the ultrahigh QY of 5.3% as octylthiophene was the first donor (CSM 7-PEG) (Fig. 9F and G). Given the highest QY, CSM 7-PEG was applied for ultrafast vasculature imaging, from which high-fidelity images were acquired (Fig. 9H). The vein and artery can be identified via principal components analysis of images from ultrafast video (Fig. 9I). This work demonstrated the effectively tunable optical properties of CSMs via molecular engineering to obtain suitable fluorophores for high-performance NIR-II imaging.

### Photoacoustic imaging

PAI is a hybrid imaging technique combining optical excitation with ultrasonic detection. This working principle endows PAI with deep penetration depth and high spatial resolution because of dramatically reduced photon scattering [109,110].

#### NIR-I PAI

Most reported CSM-based photoacoustic (PA) contrasts work for NIR-I PAI, which have received intense attention due to their advantages of strong photostability, good light-capturing ability, high photothermal conversion efficiency (PCE), and good biocompatibility. By these merits, they have been applied for diverse bioimaging.

**Fig. 10.**
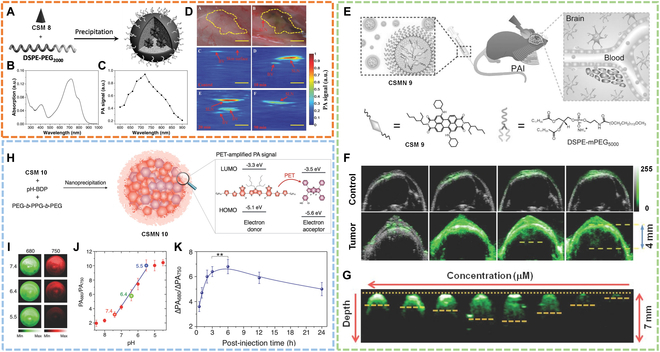
Photoacoustic imaging. (A) Schematic of CSMN 8 fabrication. (B) Absorption of CSMN 8 in water. (C) PA signal measurements at different wavelengths. (D) PA imaging of SLN. Reproduced with permission [81]. Copyright 2016, Wiley-VCH. (E) Design sketch of CSMN 9 for deep brain tumor PA imaging. (F) Deep brain tumor PA imaging at different time points after intravenous injection of CSMN 9. (G) In vivo phantom test of PA imaging at different concentrations. Reproduced with permission [82]. Copyright 2014, Wiley-VCH. (H) Schematic illustration of CSMN 10 preparation and mechanism for amplified PA signal. (I) In vitro PA imaging of CSMN 10 at different pH. (J) PA spectra of CSMN 10 at different pH. (K) Quantification of the ratio of PA signal at 680 nm to that at 750 nm. Reproduced with permission [83]. Copyright 2016, Wiley-VCH.

Real-time imaging of SLNs. For instance, Liu and colleagues [81] reported an oligomer (CSM 8) for real-time PA imaging of SLNs under 800-nm laser irradiation. Before application, CSM 8 was wrapped into nanoparticles (CSMN 8) with DSPE-PEG_2000_ via a nanoprecipitation method (Fig. 10A). The obtained nanoparticles showed good NIR absorption and strong PA signal (Fig. 10B and C). After intradermal injection, the PA signal in the SLN site peaked at 10 min and then gradually decreased, which persisted for 90 min, enabling long-time PA imaging of SLN (Fig. 10D). This work showed great promise of the oligomer nanoparticles for SLN real-time imaging.

Imaging of brain tumor. Another CSM (CSM 9) reported by Cheng’s group [82] was used for PA imaging of deep brain tumor. CSM 9 was synthesized by utilization of the tertiary amine group as a donor and the diimide group as an acceptor to form a D-π-A structure. CSM 9 was then encapsulated into water-dispersible nanoparticles (CSMN 9) with DSPE-PEG_5000_ (Fig. 10E), with an absorption peak at 700 nm. CSMN 9 was for PA imaging of the brain tumor of mice. Results showed that the deeper tumor site could be detected over time after intravenous injection of CSMN 9 (Fig. 10F). Moreover, they revealed the relationship between nanoparticles’ concentration and PAI depth (Fig. 10G), which indicated that high concentration led to lower PAI depth because most incident light was absorbed by nanoparticles on the surface so that only a little laser light could transmit to the deeper site. On the other hand, a relatively low concentration allowed more laser light to pass through the surface and reach the deeper location even though PA intensity was still weakened. This work not only developed an excellent PA contrast for achieving deep brain tumor imaging but also provided insights into improving deep PAI.

In vivo pH imaging. To further improve SNR and enhance PA intensity, Pu and colleagues [83] developed an activatable PA nanoprobe with amplified PA brightness for in vivo PH imaging, which was composed of a semiconducting oligomer (CSM 10) to emit PA signal and a dye (BODIPY, PH-BDP) to enhance PA intensity and sense PH (Fig. 10H). As PH-BDP had lower orbital energy than CSM 10, photoinduced electron transfer (PET) was favored to quench the fluorescence of CSM 10 and amplified its PA intensity. Moreover, PH-BDP possessed a hydroxy group that could be protonated under acid conditions, thus allowing pH sensing. Results showed that ratiometric PA intensity could be enhanced by about 3.1 times at different PH varying from 7.4 to 5.5 in vitro, which enabled estimation of in vivo PH (Fig. 10I to K). This work showed great potential for developing nanoprobes with high specificity and selectivity.

#### NIR-II PAI

Compared with NIR-I PAI, NIR-II PAI shows irreplaceable advantages including higher tissue penetration depth, SNR, and maximum permissible exposure (MPE) to laser [12]. However, due to the design difficulty, there is only a handful of CSMNs reported for NIR-II PAI. In PAI, the photothermal effect is naturally accompanied. For the illustration of NIR-II PAI, please refer to the first example of NIR-II PTT in the following part.

### Raman imaging

RI, as a complementary optical imaging technique, is characterized by a bio-silent region (1,800 to 2,800 cm^−1^), in which no endogenous Raman signal can be detected, enabling background-free and precise imaging [31–33]. Thus, developing CSM-based nanoprobes with prominent Raman signals in the cell-silent region of Raman is of particular significance in the field of theranostics.

#### Identification of tiny residual tumors and the margin of tumors and guidance of surgery

**Fig. 11.**
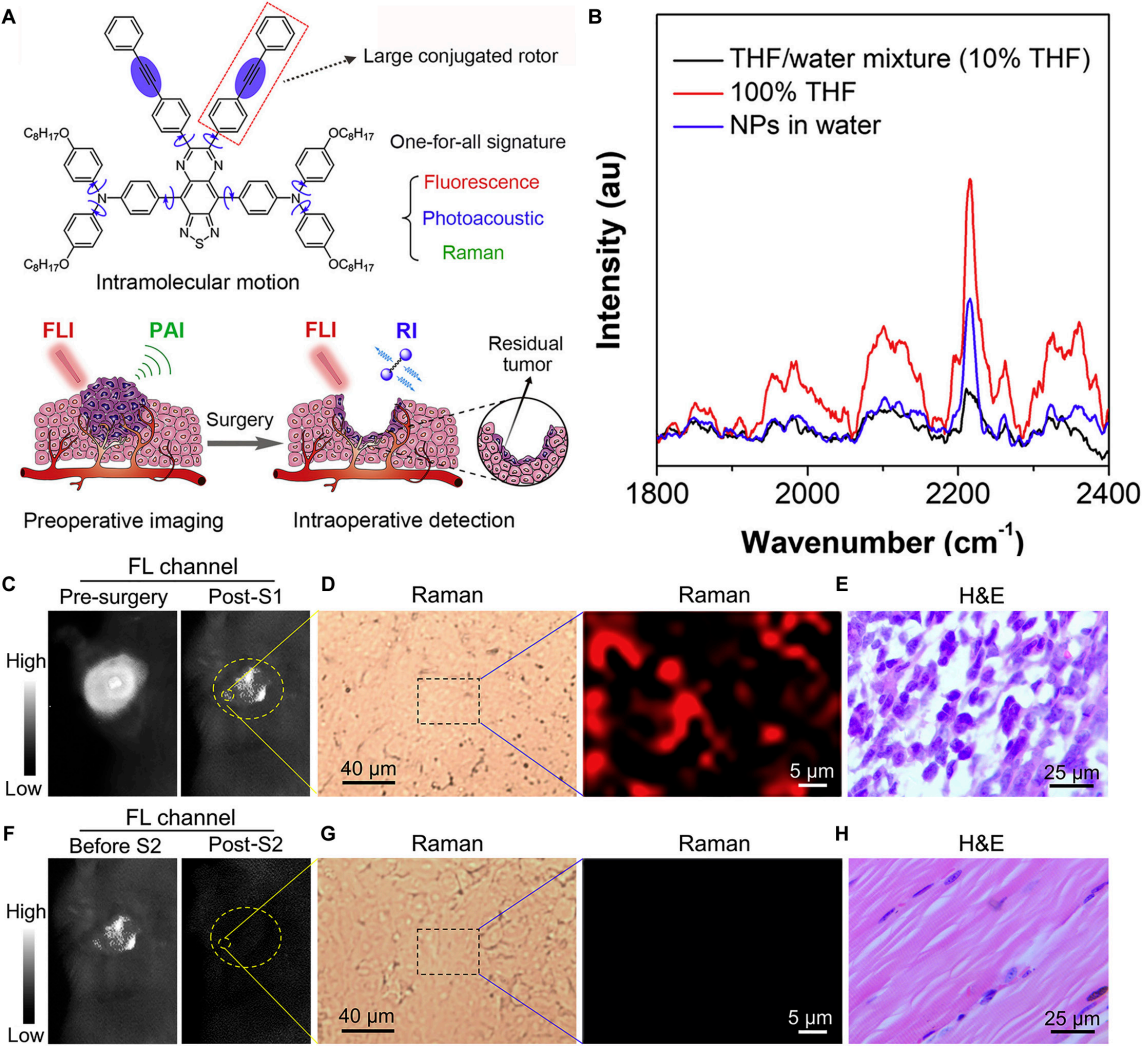
Raman imaging. (A) Scheme illustration of CSM 11 for FLI-, PAI-, and RI-guided surgery. (B) Raman signal intensity of CSM 11 in tetrahydrofuran (THF), water containing 10% THF, and nanoparticles. (C) FLI of tumor site after intravenous injection of CSMN 11 before and after first surgery (S1). (D) RI and (E) hematoxylin and eosin (H&E) staining of surgical incision site after first treatment. (F) FLI of tumor site after intravenous injection of CSMN 11 before and after second surgery (S2). (G) RI and (H) H&E staining of surgical incision site after the second treatment. Reproduced with permission [84]. Copyright 2019, Elsevier.

For example, Tang and colleagues [84] recently reported an agent (CSM 11) with boosted fluorescence, photoacoustic, and Raman signature for accurate navigation of cancer surgery (Fig. 11A). CSM-based fluorescence and PAI have been well illustrated before, so only the Raman property of CSM 11 will be highlighted here. CSM 11 with large phenyl-alkyne-phenyl substituted units displayed strong Raman signals at 2,215 cm^−1^ (Fig. 11B) in the cell-mute region. It was endowed with good water solubility by being wrapped with DSPE-PEG_2000_ to form nanoparticles (CSMN 11). Raman signal detection results showed that the unit of phenyl-alkyne-phenyl in free molecules shows higher Raman intensity than that in aggregated molecules (Fig. 11B), which might be contributed to the active intramolecular motion of phenyl-alkyne-phenyl in free molecules. CSMN 11-mediated RI was successfully applied to identify tiny residual tumors and portray the margin of tumors with microscopic resolution and high SNR during surgery (Fig. 11C to E). With the accurate guidance of intraoperative imaging, the surgeon removed the residual tiny tumors (Fig. 11F to H). After the removal of weeny tumors, the survival rate of mice was significantly improved. Therefore, this work demonstrated the great potential of CSMN-mediated RI in inspecting tiny tumors.

## Therapy

CSMNs have been widely applied in tumor therapy, including PTT and PDT, because of their favorable biocompatibility, high photostability, design flexibility, and high photon-absorbing ability. During treatment, the CSMNs first absorb incident NIR light to be excited from S_0_ to S_1_. The excited CSMs in CSMNs dissipate energy via several pathways. If the CSMs decay back to S_0_ via nonradiative decay, local heat will be generated for PTT; if they undergo ISC to T_1_, it can transfer its energy to surrounding oxygen or electrons to surrounding substrates to generate ROS for PDT. Whether PTT or PDT, they possess many merits like noninvasiveness, good efficacy, easy manipulation, and high specificity [13,111,112]. Therefore, CSMN-mediated PTT or PDT holds promise for future clinical translation in cancer therapy. Recently developed CSMNs for NIR therapy are summarized in Table 2.

### Photothermal therapy

High absorptivity and PCE are crucial for CSMNs to achieve efficient PTT [65,93]. Thus, many recent efforts have been devoted to developing and designing high-performance CSM-based NIR photothermal agents (PTAs).

#### NIR-I PTT

**Fig. 12.**
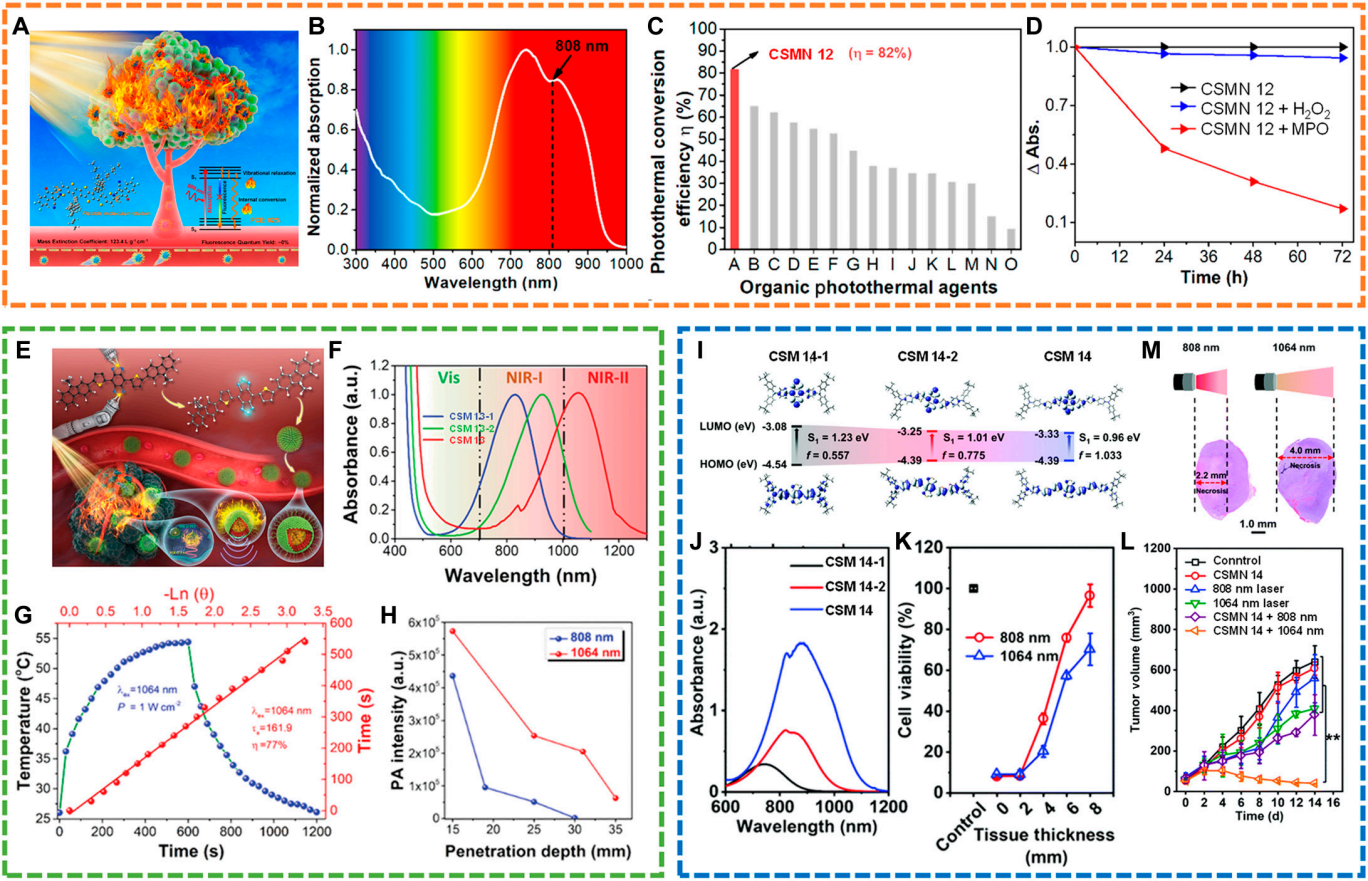
NIR-I and NIR-II PTT. (A) Schematic of CSMN 12 for highly efficient PTT. (B) Absorption spectrum of CSMN 12. (C) PCE comparison of CSMN 12 with other reported organic PTT agents. (D) Biodegradation rate of CSMN 12 treated with H_2_O_2_ or myeloperoxidase (MPO). Reproduced with permission [65]. Copyright 2019, American Chemical Society. (E) Design sketch of CSM 13 for PT. (F) Absorption spectra of CSM 13-1, CSM 13-2, and CSM 13. (G) Temperature elevating/descending and linear analysis curve of CSMN 13. (H) PA intensity change with penetration depth increase under 808- or 1,064-nm laser irradiation. Reproduced with permission [85]. Copyright 2020, Wiley-VCH. (I) Energy gaps of CSM 14, CSM 14-1, and CSM 14-2. (J) Molecular absorption spectra. (K) Cell viability evolution with increasing tissue thickness. (L) Therapy effect after different treatments. (M) H&E staining of the whole tumors after treatment with CSMN 14 under 808- or 1,064-nm laser irradiation. Reproduced with permission. Copyright 2014, Royal Society of Chemistry.

For example, our group reported an “A-D-A” structured oligomer (CSM 12) [65]. Before application, CSM 12 was transformed into water-soluble nanoparticles (CSMN 12), showing strong absorption at 808 nm and a high PCE of 82% (Fig. 12A to C). When exposed to 808-nm laser irradiation, CSMN 12 showed a fast temperature increment of around 45 °C within 5 min. After 10-cycle heating/cooling, there appeared little influence on the photothermal conversion ability of CSMN 12. The “A-D-A” structure and flexible intramolecular motion of D-A links were considered as the main reasons for the high PCE. Notably, CSMN 12 could be degraded in the bio-mimicking environment (Fig. 12D), removing the toxicity concern of CSMN 12 in living body. By strong mass extinction efficiency (123.4 l g^−1^ cm^−1^) and high PCE, CSMN 12 showed severe phototoxicity to 3 kinds of cell lines (A549, 4T1, and Hela cells). Most importantly, the tumors were thoroughly eliminated in antitumor experiments. This work thus presented an effective CSMN and paved an avenue for developing and exploring highly efficient CSMNs for PTs.

#### NIR-II PTT

Although CSMNs have received a lot of progress in NIR-I PTT, CSMN-mediated NIR-II PTT has rarely been reported because of the design difficulty of CSMs with strong absorption in the NIR-II region. Compared with NIR-I PTT, NIR-II light-initiated PTT has many advantages, such as deeper penetration depth resulting from low photon scattering and higher MPE (MPE for 1,064 nm is 1 W cm^−2^, while that for 808 nm is 0.33 W cm^−2^) [113,114]. Thus, developing and designing CSMN-based NIR-II PTT agents is highly desirable.

To achieve CSMN-mediated NIR-II PTT, our group delicately tuned the optical absorption of π-conjugated small molecules from the NIR-I window to the NIR-II window through molecular surgery of single-atom substitution (Fig. 12E) [85]. Via selenium (Se) atom introduction, an oligomer (CSM 13) with an absorption peak over 1,000 nm was first reported (Fig. 12F). After being encapsulated into nanoparticles (CSMN 13), it showed an unprecedently high PCE of 77% under 1,064-nm illumination (Fig. 12G). PA imaging showed that CSMN 13 with 1,064-nm laser irradiation showed deeper tissue penetration depth than CSMN 13-1 with 808-nm irradiation (Fig. 12H), revealing the superiority of CSMN 13 and 1,064-nm laser irradiation. Cell experiments showed that CSMN 13 could efficiently kill A549 and 4T1 cancer cells when exposed to a 1,064-nm laser. Under the guidance NIR-II PAI, in vivo PTT also achieved a satisfied curative effect, further demonstrating the excellent photothermal performance of CSMN 13. The biological evaluation indicated that CSMN 13 induced little side effects in the treated mice. Therefore, this work first realized the CSMN-mediated NIR-II PTT and offered an effective strategy for designing high-performance NIR-II CSMs.

Another kind of recently reported NIR-II small molecule for PTT application is from Ling’s group [86]. In this work, 3 conjugated molecules (CSM 14-1, CSM 14-2, and CSM 14) with typical “D-A-D” structures were designed and synthesized by adopting a molecular engineering strategy (Fig. 12I). With an increasing number of thiophen bridges, the optical absorption of these CSMs was red-shifted (Fig. 12J). When 2 thiophen units were introduced (CSM 14), the absorption simultaneously covered NIR-I and NIR-II windows. CSM 14-1, CSM 14-2, and CSM 14 were then transformed into nanoparticles (CSMN 14-1, CSMN 14-2, and CSMN 14), showing respective PCE 25.3%, 31.5, and 31.6% under 1,064-nm laser irradiation. Moreover, CSMN 14 showed a higher extinction coefficient of 5.82 l g^−1^ cm^−1^ at 1,064 nm than CSMN 14-1 (without thiophen units, 1.31 l g^−1^ cm^−1^) and CSMN 14-2 (containing one thiophen units, 0.30 l g^−1^ cm^−1^). Given its good properties, CSMN 14 was selected for the next application. Results show that CSMN 14 could achieve deeper tissue PTT under 1,064-nm laser irradiation than 808 nm both in vitro and in vivo (Fig. 12K and M), indicating the superiority of NIR-II PTT. Under 1,064-nm laser irradiation, CSMN 14 showed a good tumor ablation effect in in vivo experiments (Fig. 12L). Collectively, this contribution developed a kind of novel CSMs while proposing a useful strategy for achieving CSM-based NIR-II PTT.

### Photodynamic therapy

**Fig. 13.**
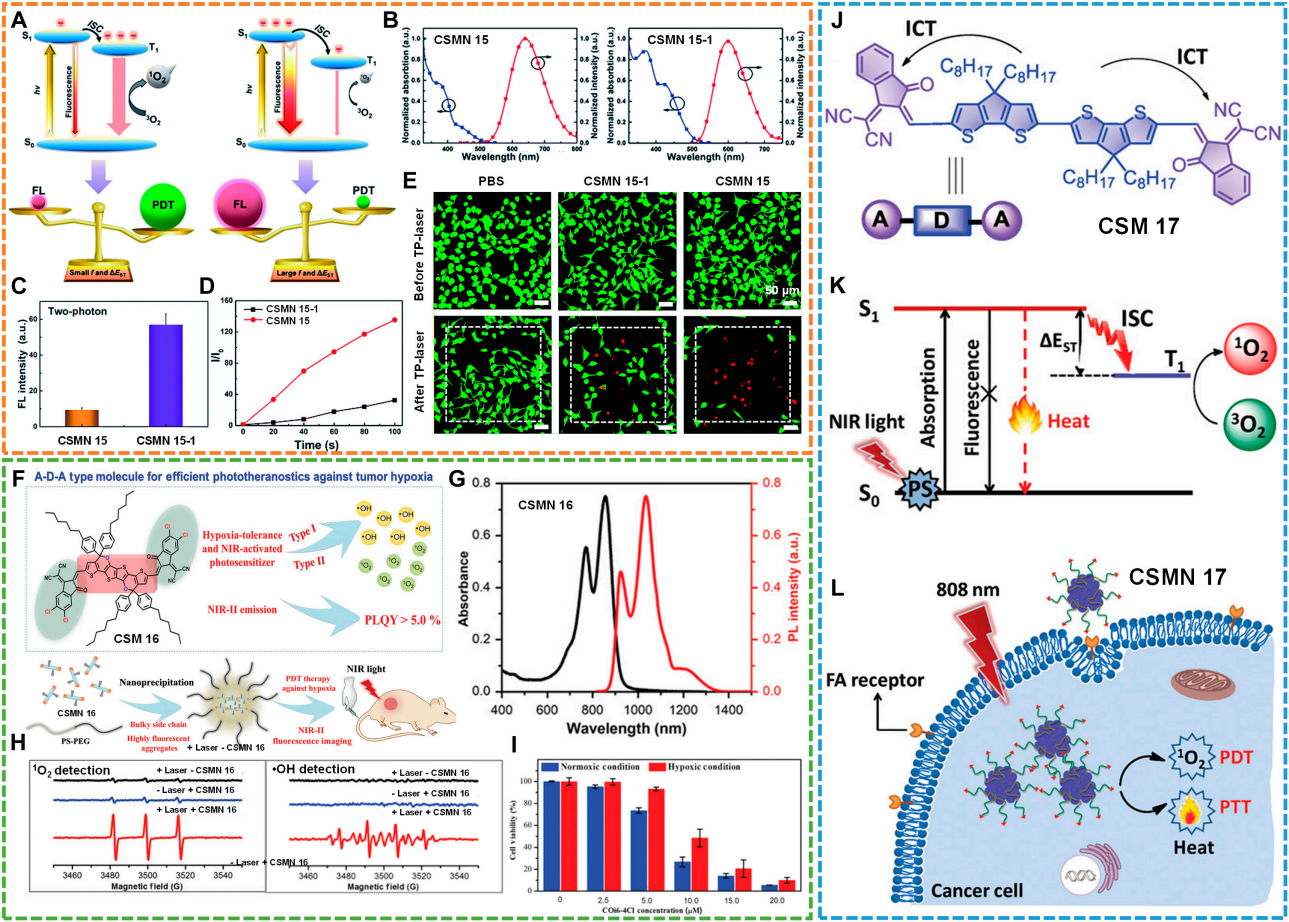
Photodynamic therapy. (A) Schematic illustration of exciton dynamics in CSM 15 and CSM 15-1. FL, fluorescence. (B) Absorption and emission spectra of CSMN 15 and CSMN 15-1. (C) Fluorescence intensities of CSMN 15 and CSMN 15-1 under 2-photon irradiation. (D) Fluorescence intensities of 2′,7′-dichlorodihydrofluorescein (DCFH) at peak wavelength dispersed in CSMN 15 or CSMN 15-1 after xenon lamp irradiation. (E) AM/PI staining of Hela cells incubated with CSMN 15 or CSMN 15-1 after irradiation with 800-nm fs laser for 200 scans. Reproduced with permission [87]. Copyright 2020, Royal Society of Chemistry. (F) Schematic of CSM 16 with bright NIR-II emission for PDT against hypoxia. (G) Absorption and emission spectra of CSMN 16. (H) ESR spectra of 2,2,6,6-tetramethylpiperidine (TEMP) probe detecting ^1^O_2_ (left) and 5,5-dimethyl-1-pyrroline N-oxide (DMPO) probe detecting •OH (right). (I) 3-(4,5)-Dimethylthiahiazo (-z-y1)-3,5-di-phenytetrazoliumromide‌ (MTT) assay of 4T1 cells incubated with CSMN 16 at different concentrations under 808-nm laser irradiation in normoxia or hypoxia conditions. Reproduced with permission [88]. Copyright 2020, Wiley-VCH. (J) Molecular structure of CSM 17. (K) Working mechanism of CSMN 17 for PDT and PTT. (L) Schematic of CSMN 17 for dual-modal phototherapy. Reproduced with permission [89]. Copyright 2020, Wiley-VCH.

Most photosensitizers (PSs) possess relatively short excitation wavelengths (400 to 700 nm), which show the limitation of shallow tissue penetration depth, hindering their further bioapplication. NIR light-excited PSs can greatly address this problem and thus are expected to be promising PDT candidates. Unfortunately, it is well known that the design and synthesis of NIR-absorbing PSs are very difficult because of their notorious instability and high triplet electronic energy requirement. To achieve NIR PDT, our group adopted NIR 2-photon excited PSs for FLI and PDT (Fig. 13A and B) [87]. In this work, we reported a guideline for designing high-performance PSs and fluorophores by regulating Δ*E*_ST_ and oscillator strength (*f*). By introducing electron donors with different electron-donating abilities, 2 synthesized molecules (CSM 15 and CSM 15-1) showed tunable Δ*E*_ST_ and *f.* CSM 15 with small Δ*E*_ST_ and *f* was beneficial for ROS generation (Fig. 13D), while CSM 15-1 with large Δ*E*_ST_ was favorable for fluorescence emission (Fig. 13C). Under 2-photon irradiation (800 nm), the assembled CSM 15 (CSMN 15) showed a more efficient PDT effect (Fig. 13E) than CSMN 15-1. This study thus provided an effective strategy for designing and developing efficient fluorescent imaging and PDT agents.

Moreover, Tian and colleagues [88] recently reported an “A-D-A”-structured PS (CSM 16) with bright NIR-II emission for efficient type-I and type-II PDT (Fig. 13F). After assembling into nanoparticles (CSMN 16), CSM 16 showed NIR absorption ranging from 700 to 1,000 nm, and NIR-II fluorescence emission peaked at 1,035 nm with a high QY of 5.2% (Fig. 13G). The electron spin resonance (ESR) measurements confirmed the generation of ROS, including ^1^O_2_ and •OH by CSMN 16 (Fig. 13H). The ROS QY of CSMN 16 was calculated to be 2.7% with ICG as reference (0.2%). Because of the concurrent generation of ^1^O_2_ and •OH, CSMN 16 efficiently killed the cancer cells (>90%) both under normoxia and hypoxia after 880-nm laser irradiation (Fig. 13I). In vivo experiments also confirmed the high-performance PDT effect of CSMN 16. Therefore, this work showed great potential for accelerating the development of efficient hypoxia-resistant NIR PSs.

Another work reported by Chen’s group [89] presented another “A-D-A”-typed PS (CSM 17) for combinational PDT and PTT (Fig. 13J to L). This designed PS showed suitable energy levels and intense ICT, enabling efficient dual-mode phototherapy. After assembling with the folate-functionalized copolymer, the prepared nanoparticles (CSMN 17) showed good biocompatibility, high photostability, ^1^O_2_ QY (18.6%), and PCE (36.5%), and active targetability to cancer cells. By these merits, CSMN 17 achieved good anticancer efficacy under 808-nm irradiation. This work thus revealed the great promise of “A-D-A”-structured single molecule as efficient PSs for NIR light-initiated dual phototherapy of tumors.

## Synergistic PT

Synergistic PTs that can simultaneously achieve diagnosis and therapy show obvious advantages by comparison with single imaging or therapy mode. With finely engineered structures and superior photophysical properties, CSMNs have received wide recent attention. The following will illustrate from 3 perspectives based on the functionalities of CSMNs: dual-function CSMNs, tri-function CSMNs, and tetra-function CSMNs. Recently developed CSMNs for NIR synergistic PTs are summarized in Table 3.

### Dual-function CSMNs

PAI-guided PTT shows many charming merits, such as noninvasiveness, easy manipulation, minimal adverse effects, and high therapy outcomes [115,116]. PTT is naturally accompanied with PAI because the PA signal is from heat-generated acoustic waves [117,118]. Thus, CSMNs in PAI and PTT can exert a maximized imaging therapy effect simultaneously and will not compromise each other. Moreover, the photo-absorbing/sound release working principle enables the PAI technique to noninvasively probe deep-tissue information (5 to 7 cm) with high spatial resolution and contrast [75,119,120]. PTT possesses many advantages, such as noninvasiveness, good therapy effect, and easy manipulation [48,121]. Thereby, PAI/PTT is one of the most promising PT modes.

**Fig. 14.**
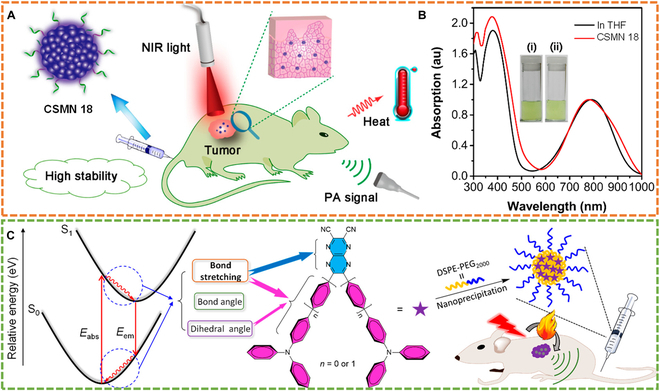
Dual-function CSMNs. (A) Schematic drawing of CSMN 18 for PAI-guided PTT. (B) Absorption spectra of CSM 18 dissolved in THF and CSMN 18 dispersed in water. Reproduced with permission [90]. Copyright 2017, American Chemical Society. (C) Diagram of motion models of CSM 19 and CSM 19-1 for PAI and PTT application. Reproduced with permission [91]. Copyright 2020, American Chemical Society.

To overcome the problem of poor photostability, photobleaching, and intolerance to reactive oxygen/nitrogen species (RONS) of NIR-absorbing organic small molecules, Tang and coworkers [90] reported a small molecule (CSM 18)-based nanoparticles (CSMN 18) with intense NIR absorption within 700 to 900 nm for highly effective PTT under the guidance of PAI (Fig. 14A and B). Compared with the conventional cyanine, ICG, CSMN 18 showed much stronger thermal/photothermal stabilities and photobleaching/RONS resistances. These merits enabled CSMN 18 to significantly suppress tumor growth in vivo under the guidance of PAI. To boost the photothermal effect and PA signal of CSMs, another work from Tang’s group [91] reported a concept of bond stretching vibration, a kind of molecular motion with high frequency and insensitive to external environmental restraint. As a proof of concept, 2 D-A structured compounds (CSM 19 and CSM 19-1) were synthesized. The only difference between these 2 molecules was that CSM 19-1 possessed 2 more phenyl groups than CSM 19, contributing to the increased bond stretching vibration in CSM 19-1 (Fig. 14C). By the increased vibration, CSMN 19-1 showed better performance than CSMN 19 regarding the PCE (59% versus 52%), PA signal generation, and PA imaging of tumors. This work thus provided a useful strategy to improve the photothermic and PA effect by utilizing intramolecular bond stretching vibration.

**Fig. 15.**
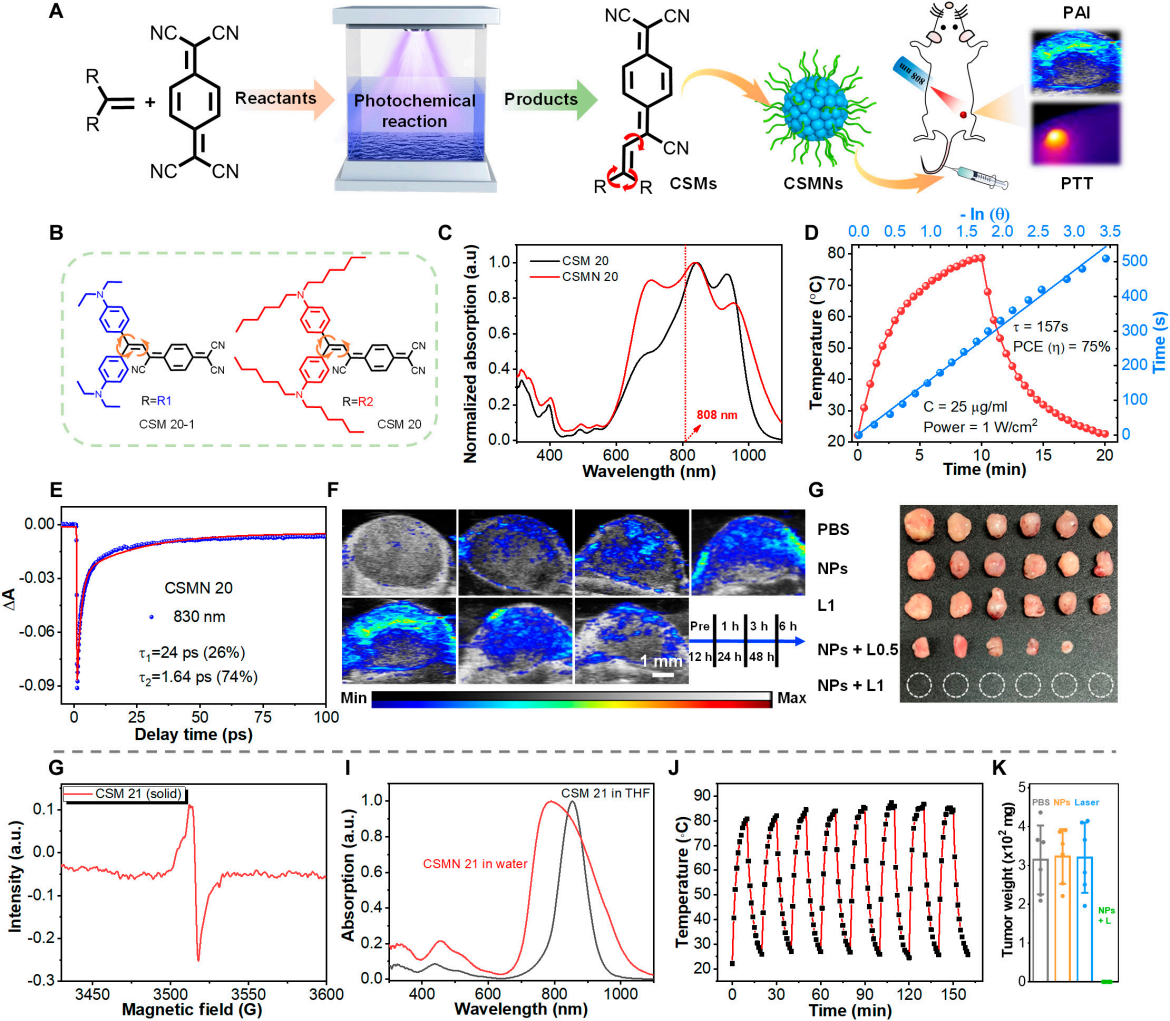
Dual-function CSMNs. (A) Schematic illustration of photochemical synthesis of CSMNs for PAI and PTT. (B) Chemical structures of CSM 20-1 and CSM 20. (C) Absorption spectra of CSM 20 and CSMN 20. (D) Temperature increase and decrease curve of CSMN 20 as well as linear fitting between time and −ln(θ). (E) Kinetic decay traces at 830 nm and corresponding fitting curves of CSMN 21. (F) In vivo PA imaging at different time points after intravenous injection of CSMN 20. (G) Images of isolated tumors after treatment. Reproduced with permission [92]. Copyright 2021, Wiley-VCH. (H) Absorption spectra of CSM 21 and CSMN 21. (I) ESR spectra of CSM 21 (solid). (J) Photostability test of CSMN 21. (K) Measurements of tumor weight in different groups. Reproduced with permission [93]. Copyright 2021, American Chemical Society.

Although numerous PTAs have been developed in recent years, most employed synthetic methods require noble metal catalysts and high temperatures. To remove these concerns, our group reported a facile photochemical reaction for synthesizing organic PTAs with no need for noble metal catalysts and heating (Fig. 15A) [92]. Through this reaction, 2 CSMs (CSM 20-1 and CSM 20) with highly nonplanar structures were first designed and synthesized (Fig. 15B). The synthesized molecules were constructed with 2 same donors (Michler’s base) and one acceptor (tricyanoquinodimethane), showing good optical absorption over 1,000 nm (Fig. 15C), remarkable NIR extinction coefficients, and excellent photothermal effect. The assembled nanoparticles (CSMN 20) showed good biocompatibility, excellent anti-photobleaching ability, and a high PCE of 75% (Fig. 15D). The excited state dynamic study further revealed the ultrafast nonradiative decay of the excited molecules in CSMN 20 (Fig. 15E). By these merits, CSMN 20 achieved high-resolution in vivo PAI (Fig. 15F) and high-efficiency photothermal tumor ablation (Fig. 15G). This work thus provided a useful approach as a supplementary to current methods for synthesizing effective CSM-based PTAs.

Reported CSMs typically possess low NIR absorbances. To boost the absorbance, our group reported a diradicaloid molecular structure (CSM 21) for highly effective PAI-guided PTT [93]. The intense charge transfer resulting from D-A interaction in CSM 21 led to an obvious diradical character (Fig. 15H), which was conducive to NIR absorption (Fig. 15I). CSM 21 possessed a much higher mass extinction coefficient of ~220 l/g/cm compared with those (~5 to 100 l/g/cm) of typical organic molecules. Assembled CSM 21 (CSMN 21) could resist long-time photoirradiation (Fig. 15J) and produce sufficient heat for PTT with a high PCE of 68%. Cell experiments showed that CSMN 21 achieved over 90% A549 cell fatality rate even at a relatively low concentration (6.25 μg/ml). In vivo anticancer experiments realized complete tumor ablation under the guidance of PAI (Fig. 15K). This work thus showed the great potential of developing diradicaloid molecules for improving NIR absorbance to facilitate biomedical applications. Except for this, other croconaine dyes have also been reported for NIR bioimaging and theranostics [122–124].

### Tri-function CSMNs

**Fig. 16.**
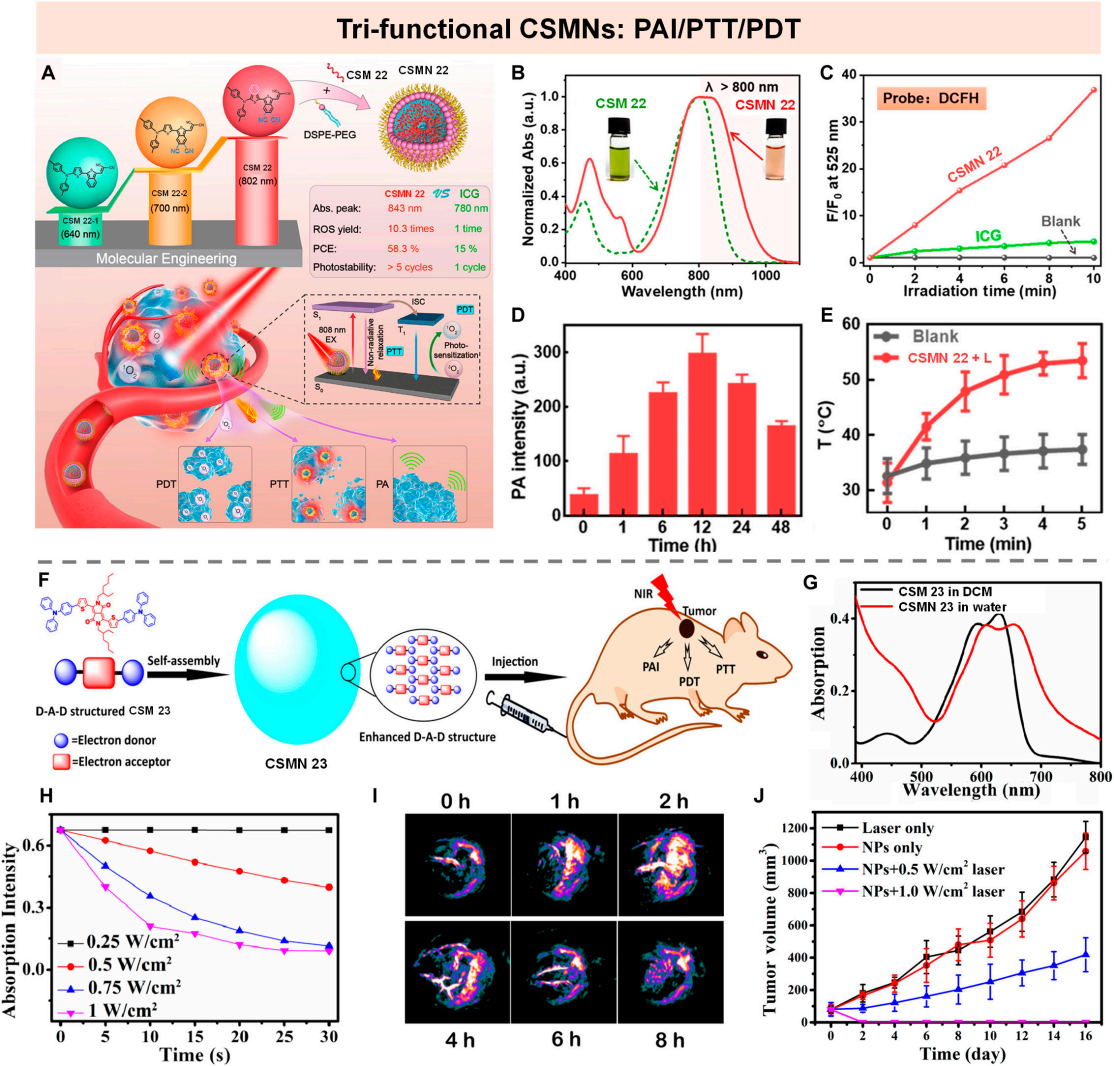
Tri-function CSMNs. (A) Chemical structures of CSM 22-1, CSM 22-2, and CSM 22 and main properties of CSMN 22 (top) as well as a schematic illustration of CSMN 22 for PAI-guided PDT and PTT (bottom). (B) Absorption spectra of CSM 22 in THF and CSMN 22 in water. (C) Fluorescence elevation rate of DCFH alone, CSMN 22, and ICG at 525 nm upon pre-exposure to an 808-nm laser for different duration time. (D) PA intensities at tumor site before and after intravenous injection of CSMN 22 at different time points. (E) Temperature increase curves of PBS (Blank) and CSMN 22 under laser irradiation (CSMN 22 + L). Reproduced with permission [94]. Copyright 2020, American Chemical Society. (F) Schematic of CSMN 23 self-assembled from D-A-D structured CSM 23 for PAI-guided PDT and PTT. (G) Absorption spectra of CSM 23 in dichloromethane (DCM) and CSMN 23 in water. (H) Absorption intensity evolution curve at 418 nm of CSM 23 mixed with 1,3-diphenylisobenzofuran (DPBF) in DCM under 660-nm laser irradiation with different power densities. (I) PA imaging at tumor site at different time points. (J) Tumor suppression curves in different groups. Reproduced with permission [95]. Copyright 2017, American Chemical Society.

NIR light-excited PTs are highly desirable because of their high penetration depth. However, their further development is greatly hindered by a limited number of PSs with long-wavelength absorption. To break this deadlock, our group reported a series of benzo[c]-thiophene (BT)-based molecules (CSM 22-1, CSM 22-2, and CSM 22) with tunable NIR absorption by modulating the electronic characters of donor and/or π subunits for multimodal PTs (Fig. 16A) [94]. Among these 3 molecules, CSM 22, with the maximum absorption peak at 802 nm, was prepared into nanoparticles (CSMN 22) (Fig. 16B). Remarkably, the ROS generation ability of CSMN 22 was 10.3-fold higher than ICG (Fig. 16C). Moreover, upon exposure to an 808-nm laser, CSMN 22 exhibited a good in vivo photoacoustic signal (Fig. 16D) and photothermal effect (Fig. 16E). With these merits, CSMN 22 achieved good PA imaging performance and dual-modal phototherapy effect.

Another small molecule (CSM 23) for PAI-guided PDT/PTT synergistic therapy (Fig. 16F) was reported by Dong and colleagues [95]. CSM 23 was synthesized by conjugating a typical donor of TPA to a diketopyrrolopyrrole (DPP) core, in which the thiophene group in DPP was used to increase the ISC ability via the heavy atom effect, while TPA was introduced for red shifting the absorption and enhancing the charge transport capacity. CSM 23 showed a maximum absorption peak at 630 nm, while the assembled nanoparticles (CSMN 23) showed a red-shifted and widened absorption peaking at 660 nm (Fig. 16G). Moreover, CSMN 23 showed a relatively high PCE of 34.5% and singlet oxygen (^1^O_2_) generation (Φ = 33.6%) upon exposure to 660-nm laser irradiation (Fig. 16H). These advantages allowed CSMN 23 to achieve excellent tumor-elimination ability via photothermal and photodynamic synergistic effect in living subjects even at a low dosage of 0.2 mg/kg under the guidance of PAI (Fig. 16I and J).

**Fig. 17.**
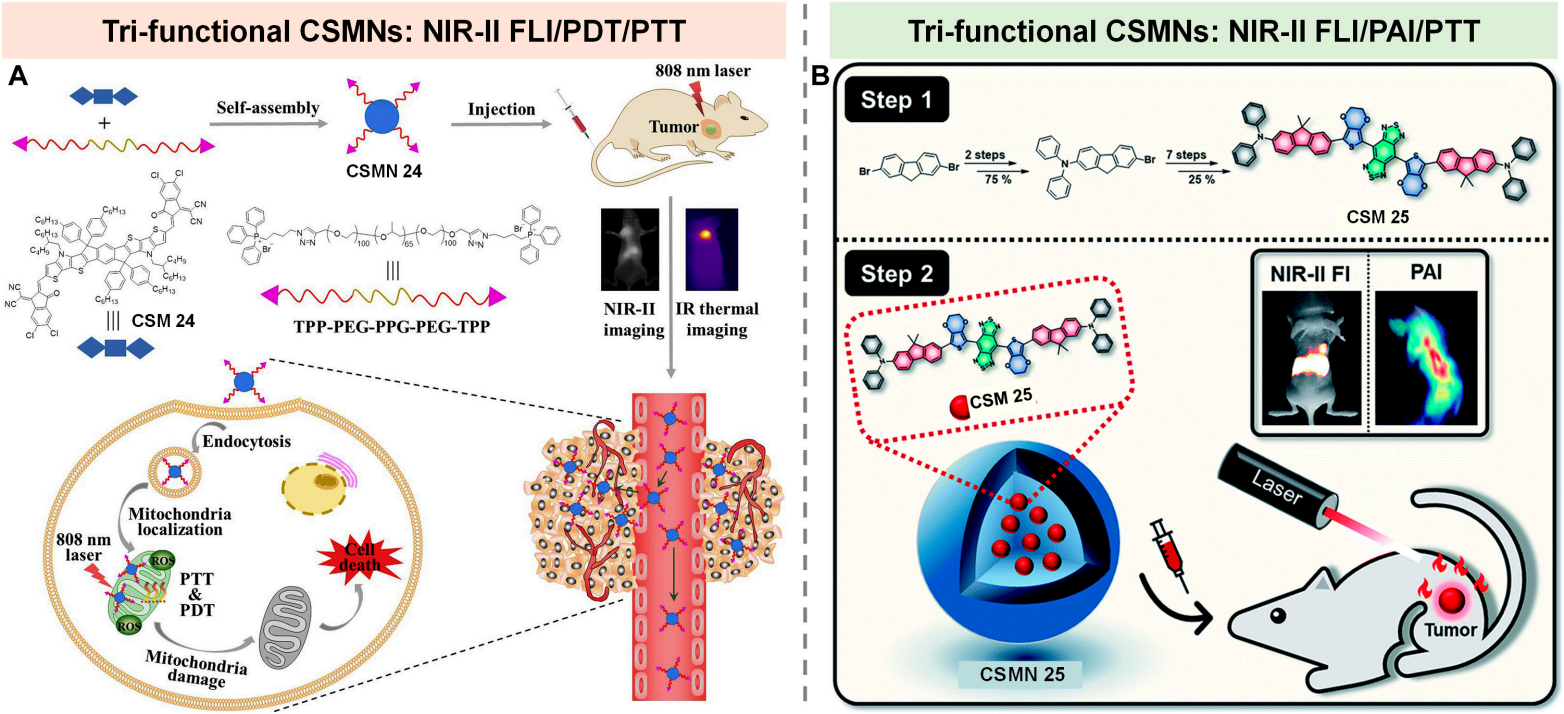
Tri-function CSMNs. (A) Schematic illustration of the preparation of CSMN 24 for NIR-II FLI, PDT, and PTT application. Reproduced with permission [96]. Copyright 2020, Elsevier. (B) Schematic illustration of the synthesis of CSMN 25 for multifunctional applications, including NIR-II FLI, PAI, and PTT. Reproduced with permission [97]. Copyright 2019, Royal Society of Chemistry.

To overcome the existing problems, such as complicated architecture and unwanted side effects in current PTs, Fan and colleagues [96] reported a one-for-all nanoplatform (CSMN 24) with mitochondria-targeting ability based on the single molecule (CSM 24) for NIR-II FLI, PDT, and PTT (Fig. 17A). CSMN 24 was formed by encapsulating hydrophobic CSM 24 using amphiphilic TPP-PEG-PPG-PEG-TPP copolymer, in which TPP functioned as a mitochondria-targeting group. Upon exposure to an 808-nm laser, CSMN 24 showed a high QY of 2.2% in water, PCE of 39.6%, and Φ of 2.3% (12 times higher than ICG). Taking these merits together, CSMN 24 achieved efficient NIR-II FLI-guided mitochondria-targeting phototherapy against tumors.

Multi-modality imaging-guided cancer therapy combining precise diagnosis and efficient treatment has become a powerful PT platform. However, currently reported multifunctional systems usually comprised multicomponent and complicated structures, which retarded the progress of clinical translation. To simplify the multifunctional system, Sun et al. reported an effective small molecule (CSM 25) with an intrinsic multifunctionality for NIR-II FLI, PAI, and PTT (Fig. 17B) [97]. The assembled nanoparticles (CSMN 25) showed bright NIR-II fluorescence that peaked at 976 nm, strong PA signals in the tumor site, and a significant tumor inhibition effect, revealing the great potential of CSMN 25 as a PT platform.

### Tetra-function CSMNs

Multifunctional PTs have recently attracted wide research interest due to their impressive synergetic effects [125–127]. However, current nanoplatforms, especially for those involving more than 3 functions, are confronted with their inherent drawbacks, such as complicated components and the requirement of multi-laser excitation, which severely hinder their further progress in clinical translation [50,84,95,128]. To solve these problems, Fan and colleagues [98] reported a single small molecule (CSM 26)-based multifunctional PT (CSMN 26), which could emit NIR-II fluorescence (0.52%), release toxic ROS (^1^O_2_ QY = 49.3%), and produce hyperthermia (PCE = 23%) upon exposure to single NIR laser (660 nm) irradiation. By these merits, CSMN 26 achieved good performance in NIR-II FLI, PAI, PTT, and PDT.

**Fig. 18.**
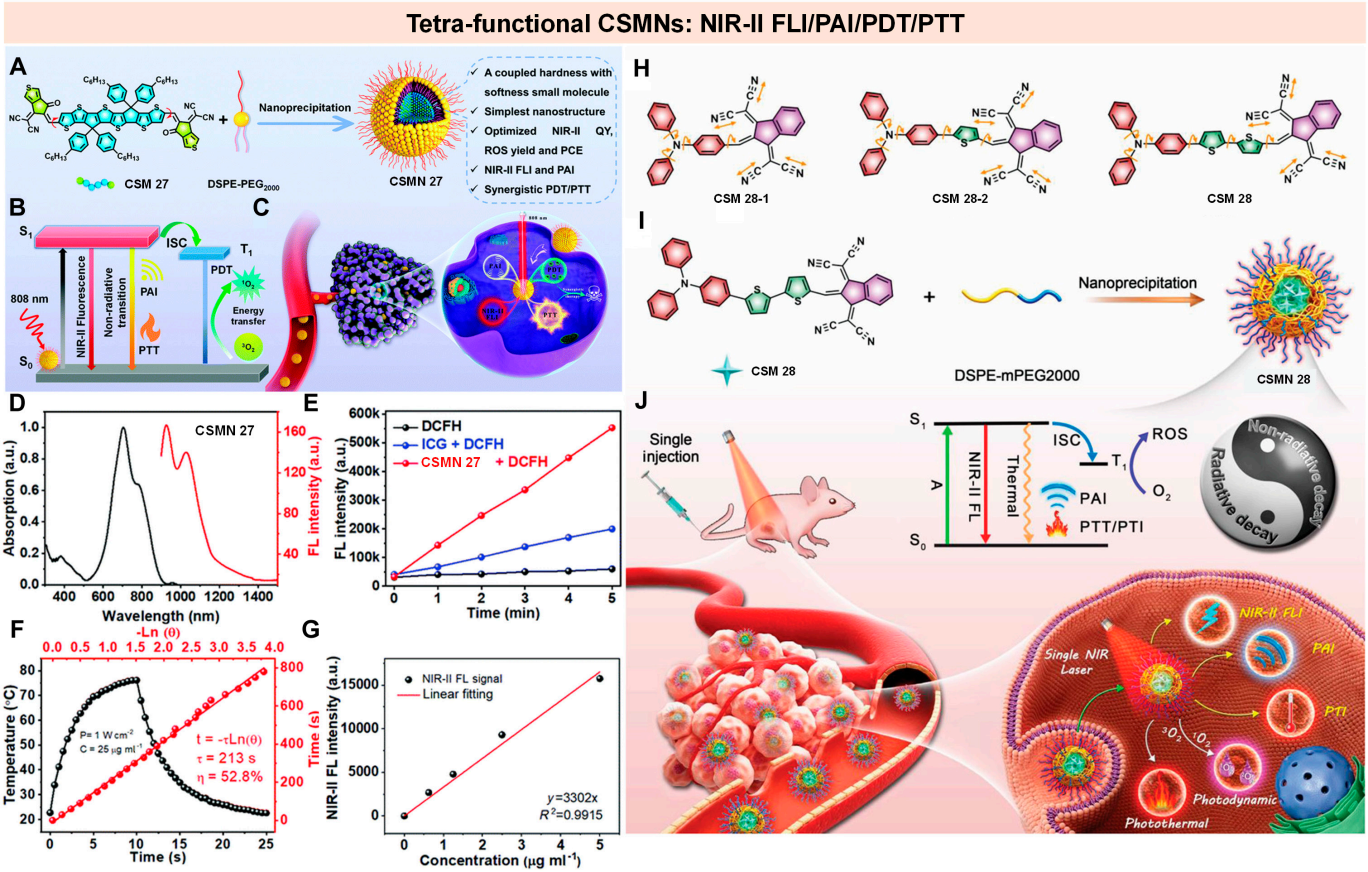
Tetra-function CSMNs. (A) Schematic illustration of the fabrication of CSMN 27 and its main properties. (B) Photophysical mechanism of CSMN 27 for NIR-II FLI, PAI, PDT, and PTT. (C) Schematic illustration of CSMN 27 for multifunctional cancer theranostics. (D) Absorption and emission spectra of CSMN 27. (E) Fluorescence intensity evolution curves of DCFH at 525 nm mixed with ICG or CSMN 27 with increasing irradiation time. (F) Temperature increase and decrease curves, and linear fitting between cooling times and −ln(θ) (θ: temperature driving force). (G) Linear fitting between NIR-II FL intensity and concentrations. Reproduced with permission [72]. Copyright 2021, Royal Society of Chemistry. (H) Chemical structures of CSM 28-1, CSM 28-2, and CSM 28. (I) Schematic of preparation of CSMN 28. (J) Illustration of photophysical processes of CSMN 28 and their applications for NIR-II FLI, PAI, PDT, and PTT. Reproduced with permission [73]. Copyright 2020, Wiley-VCH.

To integrate tetra-functions into one single molecule while balancing and maximizing the efficacy of each function, our group reported an A-D-A-structured small molecule (CSM 27) coupled with rigidness and flexibility to simultaneously achieve NIR-II FLI, PAI, PDT, and PTT (Fig. 18A to C) [72]. The assembled nanoparticles (CSMN 27) showed high NIR absorption ranging from 600 to 900 nm (Fig. 18D, left) and strong fluorescence from 900 to 1,200 nm under 808-nm laser excitation (Fig. 18D, right). CSMN 27 also displayed good singlet oxygen generation ability (3.2-fold higher than ICG) (Fig. 18E), excellent PCE (52.8%) (Fig. 18F), and high NIR-II QY (3.0%) (Fig. 18G) under 808-nm laser illumination. Such balanced functionality might be attributed to the rigid and flexible structures of CSM 27 to tactically manipulate the energy dissipation paths (nonradiative against radiative decay). Both in vitro and in vivo experiments demonstrated excellent therapeutic effects through photodynamic and photothermal synergistic therapy with the guidance of dual-mode NIR-II FLI and PAI. This work thus opened a new avenue for the design and development of single conjugated oligomer nanomaterials for versatile cancer nanomedicines.

Another example from Tang and coworkers [73] reported a simple but powerful one-for-all PTs based on aggregation-induced emission (AIE)-active fluorophores. In this work, 3 fluorophores (CSM 28-1, CSM 28-2, and CSM 28) were designed and constructed by introducing the TPA unit as donor, and/or thiophene segment as donor and π-bridge, as well as 1,3-bis(dicyanomethylidene)indane moiety as acceptor (Fig. 18H). The TPA unit not only functioned as molecular rotors, its propeller-like highly twisted conformation, but also maintained both heat generation and fluorescence, while the vigorous stretching vibrations of C≡N in 2 malononitrile-modified indanes provided extra intramolecular motion in the aggregate state. The thiophene unit in CSM 28-2 and CSM 28 was for red-shifting the absorption/emission wavelength. Such structural features endowed the AIE molecule-based nanoparticles (CSMN 28-1, CSMN 28-2, and CSMN 28) with bright NIR-II fluorescence, efficient ROS production, and significant temperature increases under 660-nm laser irradiation. Among these 3 molecules, CSMN 28 showed strong NIR fluorescence, with almost half of the emission spectrum falling in the NIR-II region (over 1,000 nm), high PCE of 46.0%, and superior ROS generation efficiency. With these merits, CSMN 28 achieved effective PAI and NIR-II FLI-guided photothermal and photodynamic synergistic therapy with only a single injection and irradiation in living mice (Fig. 18I and J). This work provided new perspectives for the design and development of versatile cancer PT.

## Conclusion and Outlook

NIR PTs, combining timely diagnosis and precise therapy in the NIR region, have attracted more and more recent attention. Compared with PTs in the UV and visible regions, NIR PTs display higher tissue penetration depth, SNR, and better biocompatibility. NIR PT agents are usually inorganic or organic. Inorganic agents are always confronted with potential biotoxicity induced by heavy metal ions and nonbiodegradability, while organic agents successfully escape these problems by their potential biodegradability, favorable optical properties, and low dark toxicity. CSMNs prepared with D-A-typed photoactive CSMs show higher photostability and tumor accumulation than cyanine dyes and more accurate molecular structures and higher reliability than SPNs. Via rational structural design, CSMs can satisfy specific requirements of NIR PTs. Such design flexibility of CSMs has permitted CSMNs to achieve more possibilities in biological applications, such as imaging (cells, tumors, lymphatic, lymph nodes, brain tumors, hindlimb vasculature, PH, vein and artery, and surgery guidance), therapy (PTT and PDT), and synergistic PTs (dual-function, tri-function, and tetra-function PTs). To exert functions, CSMNs always involve 2 mechanisms. The first one is closely connected with the Jablonski diagram, while the second one is dependent on light–tissue interactions. Except for the illustration of 2 working mechanisms, strategies for improving performances and red-shifting absorption and emission spectra of CSMNs are also highlighted.

Despite rapid development, some challenges remain in the field of CSMN-mediated NIR PTs to address. The visible or NIR-I light hardly irradiates NIR PT agents in tumors or infections in deep tissues because of limited penetration depth (several centimeters depending on tissues) [129]. As Nikola Tesla said: “If you want to know the secrets of the universe, think in terms of energy, frequency, and vibration.” Thus, developing NIR-II light-responsive CSMNs is an effective method to improve tissue penetration depth attributed to its lower photoscattering and higher MPE (MPE for 1,064 nm is 1 W cm^−2^, while that for 808 nm is 0.33 W cm^−2^). As aforementioned, our group first delicately tuned the optical absorption of π-conjugated small molecules from the NIR-I window to the NIR-II window through molecular surgery of single-atom substitution [85]. Besides, to further boost the penetration depth, other tissue penetration energy, such as ultrasound and x-ray, should be explored to irradiate CSMNs. Specifically, ultrasound, a nonionizing radiation with no known risk, has been widely applied in imaging in clinics, which can pass through 3- to 5-cm-deep tissues at a frequency of 1 MHz [130]. On the other hand, x-ray is a high-energy radiation potentially used as a good signal inducer because of its deep-tissue penetration (the whole body) and well-established application technology [131]. As reported, organic luminophores emitting NIR afterglow and producing ^1^O_2_ have realized effective cancer theranostics after x-ray irradiation [132]. Moreover, organic phosphorescent nanoscintillator have achieved low-dose x-ray-induced PDT [133]. Thus, introducing ultrasound and x-ray as tissue penetration energy to CSMNs is innovative but dependent on rational molecule design.

The performances of CSMNs, involving their absorption, fluorescence emission, photothermal conversion,^1^O_2_ generation, and in vitro and in vivo PT efficacies, can be evaluated by extinction coefficients (𝜺), fluorescence QYs (Φ_f_), PCE, ^1^O_2_ QYs (Φ), cell viabilities, and tumor volume–time curves, respectively. According to the summarized information in Tables 1 to 3, the absorption, excitation, and emission wavelengths of CSMNs and CSM-PEG range from 300 to 1,100 nm, 660 to 800 nm, and 600 to 1,200 nm, respectively. Their PCE, Φ_f_, and Φ locate within 21.8% and 82%, 0.2% and 5.3% (NIR-II, in water), and 0.61% and 33.6%, separately. Remarkably, there is much room for CSMNs to improve regarding NIR-II FL QYs and ^1^O_2_ QY. To overcome this challenge, the PT agents should maximize their absorption upon exposure to a laser. Moreover, their energy dissipation paths, including nonradiative decay, radiative decay, and ISC, should be manipulated tactically via rational molecular design. Most importantly, the performances of CSMs should cater to clinical requirements.

Currently reported structure types include D-A-D, A-D-A, S-D-A-D-S, D-π-A, D-π-A-π-D, D-A, DD-A-DD, and D-π-π-A. By arranging electron donors and acceptors rationally, the CSMs can be equipped with the desired properties to achieve effective NIR PTs. It should be mentioned that only 2 categories of CSMs with similar acceptors have been reported for NIR-II PAI and PTT, which is owing to the design difficulty of CSMs with strong absorption in the NIR-II region. Particularly, most CSMs utilize electron-deficient benzo[1,2-c:4,5-c0]bis([1,2,5]thiadiazole) (BBTD) as their acceptor because of its strong electron-withdrawing ability. With the introduction of different donors, various molecules can be designed and synthesized for different purposes. In this review, enhancing the performance and shifting the absorption and emission spectra to longer wavelengths are key problems to address, closely dependent on effective molecular engineering and nanomodulation strategy.

Light-responsive CSMNs are well elaborated in this review. However, except for light, another external stimulus (such as ultrasound, x-ray, magnet, electricity, and heat as supplementary powerful energy sources)-responsive CSMNs should be further exploited to widen the applications of CSMNs. Besides, internal stimuli (such as redox, enzyme, PH, and temperature) should be taken into consideration when designing smart CSMs, which could diversify the applications of CSMNs.

Biosafety problems are closely associated with clinic translation. Exploring, developing, and designing biodegradable or renal-clearable CSMs are effective strategies to improve biosafety. Moreover, encapsulating CSMs with amphiphilic polymers (such as DSPE-PEG_2000_, DSPE-PEG_5000_-folate, DSPE-PEG_5000_, PEG-b-PPG-b-PEG, PS-b-PEG, and TPP-PEG-PPG-PEG-TPP) or modifying CSMs with hydrophilic molecules is a commonly used method to endow CSMs with good water solubility, targetability, and biocompatibility.

Currently, most reported CSMNs are applied at only the level of small animals such as mice. Applying them to large animals or nonhuman primates would be beneficial to accelerate their clinical translation. However, the development of experiments in nonhuman primates and the translation of these experiments to clinical human experiments face many challenges, such as the following: (a) The differences in body structure and internal circulation between nonhuman primates and small animals, and whether the experimental results in mice can achieve the same effect in nonhuman primates. (b) High cost: Nonhuman primates used for preclinical experiments are usually expensive, and there are financial limitations in using nonhuman primates for experiments before the perfect effect of the CSMNs is realized. (c) Safety regulation: The clinical transformation of CSMNs usually needs to invest a lot of work in the early stage to explore their possible toxic side effects and their impact on the human body. The small animal experiments are not enough to fully reflect the toxic side effects of CSMNs entering the human body. With rapid progress in chemistry, material science, photonics, nanotechnology, and nanomedicine, these challenges will be readily resolved in the coming future.

## References

[1] Ng KK, Zheng G. Molecular interactions in organic nanoparticles for phototheranostic applications. Chem Rev. 2015;115(19):11012–11042. 26244706 10.1021/acs.chemrev.5b00140

[2] Chen H, Zhang W, Zhu G, Xie J, Chen X. Rethinking cancer nanotheranostics. Nat Rev Mater. 2017;2:17024. 29075517 10.1038/natrevmats.2017.24 PMC5654564

[3] Li C. A targeted approach to cancer imaging and therapy. Nat Mater. 2014;13(2):110–115. 24452345 10.1038/nmat3877 PMC4234179

[4] Vankayala R, Hwang KC. Near-infrared-light-activatable nanomaterial-mediated phototheranostic nanomedicines: An emerging paradigm for cancer treatment. Adv Mater. 2018;30(23):Article e1706320. 29577458 10.1002/adma.201706320

[5] Yang T, Wang Y, Ke H, Wang Q, Lv X, Wu H, Tang Y, Yang X, Chen C, Zhao Y, et al. Protein-nanoreactor-assisted synthesis of semiconductor nanocrystals for efficient cancer theranostics. Adv Mater. 2016;28(28):5923–5930. 27165472 10.1002/adma.201506119

[6] Feng G, Liu B. Aggregation-induced emission(AIE) dots: Emerging theranostic nanolights. Acc Chem Res. 2018;51(6):1404–1414. 29733571 10.1021/acs.accounts.8b00060

[7] Cheng Z, Al Zaki A, Hui JZ, Muzykantov VR, Tsourkas A. Multifunctional nanoparticles: Cost versus benefit of adding targeting and imaging capabilities. Science. 2012;338(6109):903–910. 23161990 10.1126/science.1226338 PMC3660151

[8] Zhao X, Yang C-X, Chen L-G, Yan X-P. Dual-stimuli responsive and reversibly activatable theranostic nanoprobe for precision tumor-targeting and fluorescence-guided photothermal therapy. Nat Commun. 2017;8:14998. 28524865 10.1038/ncomms14998 PMC5454460

[9] Zhang J, Chen J, Ren J, Guo W, Li X, Chen R, Chelora J, Cui X, Wan Y, Liang X-J, et al. Biocompatible semiconducting polymer nanoparticles as robust photoacoustic and photothermal agents revealing the effects of chemical structure on high photothermal conversion efficiency. Biomaterials. 2018;181:92–102. 30081305 10.1016/j.biomaterials.2018.07.042

[10] Ai X, Ho CJH, Aw J, Attia ABE, Mu J, Wang Y, Wang X, Wang Y, Liu X, Chen H, et al. In vivo covalent cross-linking of photon-converted rare-earth nanostructures for tumour localization and theranostics. Nat Commun. 2016;7:10432. 26786559 10.1038/ncomms10432 PMC4736106

[11] Li M, Long S, Kang Y, Guo L, Wang J, Fan J, Du J, Peng X. De novo design of phototheranostic sensitizers based on structure-inherent targeting for enhanced cancer ablation. J Am Chem Soc. 2018;140(46):15820–15826. 30380856 10.1021/jacs.8b09117

[12] Yin C, Li X, Wang Y, Liang Y, Zhou S, Zhao P, Lee CS, Fan Q, Huang W. Organic semiconducting macromolecular dyes for NIR-II photoacoustic imaging and photothermal therapy. Adv Funct Mater. 2021;31(37):2104650.

[13] Yang T, Liu L, Deng Y, Guo Z, Zhang G, Ge Z, Ke H, Chen H. Ultrastable near-infrared conjugated-polymer nanoparticles for dually photoactive tumor inhibition. Adv Mater. 2017;29(31):1700487. 10.1002/adma.201700487 28626897

[14] Yang K, Xu H, Cheng L, Sun C, Wang J, Liu Z. In vitro and in vivo near-infrared photothermal therapy of cancer using polypyrrole organic nanoparticles. Adv Mater. 2012;24(41):5586–5592. 22907876 10.1002/adma.201202625

[15] Hang Y, Boryczka J, Wu N. Visible-light and near-infrared fluorescence and surface-enhanced Raman scattering point-of-care sensing and bio-imaging: A review. Chem Soc Rev. 2022;51(1):329–375. 34897302 10.1039/c9cs00621d PMC9135580

[16] Li J, Duan H, Pu K. Nanotransducers for near-infrared photoregulation in biomedicine. Adv Mater. 2019;31(33):Article e1901607. 31199021 10.1002/adma.201901607

[17] Choi HS, Gibbs SL, Lee JH, Kim SH, Ashitate Y, Liu F, Hyun H, Park G, Xie Y, Bae S, et al. Targeted zwitterionic near-infrared fluorophores for improved optical imaging. Nat Biotechnol. 2013;31(2):148–153. 23292608 10.1038/nbt.2468 PMC3568187

[18] Schmidt EL, Ou Z, Ximendes E, Cui H, Keck CHC, Jaque D, Hong G. Near-infrared II fluorescence imaging. Nat Rev Methods Primers. 2024;4:23.

[19] Zhang Y-Q, Liu W-L, Luo X-J, Shi J-P, Zeng Y-Z, Chen W-L, Huang W-H, Zhu Y-Y, Gao W-L, Li R-H, et al. Novel self-assembled multifunctional nanoprobes for second-near-infrared-fluorescence-image-guided breast cancer surgery and enhanced radiotherapy efficacy. Adv Sci. 2023;10(10):e2205294. 10.1002/advs.202205294 PMC10074043 36721054

[20] Schäferling M. The art of fluorescence imaging with chemical sensors. Angew Chem Int Ed Engl. 2012;51(15):3532–3554. 22422626 10.1002/anie.201105459

[21] Kang H, Shamim M, Yin X, Adluru E, Fukuda T, Yokomizo S, Chang H, Park SH, Cui Y, Moy AJ, et al. Tumor-associated immune-cell-mediated tumor-targeting mechanism with NIR-II fluorescence imaging. Adv Mater. 2022;34(8):e2106500. 34913533 10.1002/adma.202106500 PMC8881361

[22] Gioux S, Choi HS, Frangioni JV. Image-guided surgery using invisible near-infrared light: Fundamentals of clinical translation. Mol Imaging. 2010;9(5):237–255. 20868625 PMC3105445

[23] Hong G, Lee JC, Robinson JT, Raaz U, Xie L, Huang NF, Cooke JP, Dai H. Multifunctional in vivo vascular imaging using near-infrared II fluorescence. Nat Med. 2012;18:1841–1846. 23160236 10.1038/nm.2995 PMC3595196

[24] Lin L, Wang LV. The emerging role of photoacoustic imaging in clinical oncology. Nat Rev Clin Oncol. 2022;19:365–384. 35322236 10.1038/s41571-022-00615-3

[25] Choi W, Park B, Choi S, Oh D, Kim J, Kim C. Recent advances in contrast-enhanced photoacoustic imaging: Overcoming the physical and practical challenges. Chem Rev. 2023;123(11):7379–7419. 36642892 10.1021/acs.chemrev.2c00627

[26] Zhao Z, Swartchick CB, Chan J. Targeted contrast agents and activatable probes for photoacoustic imaging of cancer. Chem Soc Rev. 2022;51(3):829–868. 35094040 10.1039/d0cs00771d PMC9549347

[27] Zhang W, Chan C, Zhang K, Qin H, Yu B-Y, Xue Z, Zheng X, Tian J. Discovering a new drug against acute kidney injury by using a tailored photoacoustic imaging probe. Adv Mater. 2024;36(18):e2311397. 38221651 10.1002/adma.202311397

[28] Pu K, Shuhendler AJ, Jokerst JV, Mei J, Gambhir SS, Bao Z, Rao J. Semiconducting polymer nanoparticles as photoacoustic molecular imaging probes in living mice. Nat Nanotechnol. 2014;9(3):233–239. 24463363 10.1038/nnano.2013.302 PMC3947658

[29] Zhang Y, Jeon M, Rich LJ, Hong H, Geng J, Zhang Y, Shi S, Barnhart TE, Alexandridis P, Huizinga JD, et al. Non-invasive multimodal functional imaging of the intestine with frozen micellar naphthalocyanines. Nat Nanotechnol. 2014;9(8):631–638. 24997526 10.1038/nnano.2014.130 PMC4130353

[30] Jathoul AP, Laufer J, Ogunlade O, Treeby B, Cox B, Zhang E, Johnson P, Pizzey AR, Philip B, Marafioti T, et al. Deep in vivo photoacoustic imaging of mammalian tissues using a tyrosinase-based genetic reporter. Nat Photonics. 2015;9:239–246.

[31] Wei L, Hu F, Chen Z, Shen Y, Zhang L, Min W. Live-cell bioorthogonal chemical imaging: Stimulated Raman scattering microscopy of vibrational probes. Acc Chem Res. 2016;49(8):1494–1502. 27486796 10.1021/acs.accounts.6b00210 PMC5704954

[32] Song Z-L, Chen Z, Bian X, Zhou L-Y, Ding D, Liang H, Zou Y-X, Wang S-S, Chen L, Yang C, et al. Alkyne-functionalized superstable graphitic silver nanoparticles for Raman imaging. J Am Chem Soc. 2014;136(39):13558–13561. 25233109 10.1021/ja507368z PMC4183632

[33] Li S, Chen T, Wang Y, Liu L, Lv F, Li Z, Huang Y, Schanze KS, Wang S. Conjugated polymer with intrinsic alkyne units for synergistically enhanced Raman imaging in living cells. Angew Chem Int Ed Engl. 2017;56(43):13455–13458. 28851103 10.1002/anie.201707042

[34] Nguyen V-N, Zhao Z, Tang BZ, Yoon J. Organic photosensitizers for antimicrobial phototherapy. Chem Soc Rev. 2022;51(9):3324–3340. 35373787 10.1039/d1cs00647a

[35] Yun SH, Kwok SJJ. Light in diagnosis, therapy and surgery. Nat Biomed Eng. 2017;1:0008. 28649464 10.1038/s41551-016-0008 PMC5476943

[36] Li J, Pu K. Development of organic semiconducting materials for deep-tissue optical imaging, phototherapy and photoactivation. Chem Soc Rev. 2019;48(1):38–71. 30387803 10.1039/c8cs00001h

[37] Liang C, Xu L, Song G, Liu Z. Emerging nanomedicine approaches fighting tumor metastasis: Animal models, metastasis-targeted drug delivery, phototherapy, and immunotherapy. Chem Soc Rev. 2016;45(22):6250–6269. 27333329 10.1039/c6cs00458j

[38] Dolmans DEJGJ, Fukumura D, Jain RK. Photodynamic therapy for cancer. Nat Rev Cancer. 2003;3(5):380–387. 12724736 10.1038/nrc1071

[39] Li T, Liu L, Xu P, Yuan P, Tian Y, Cheng Q, Yan L. Multifunctional nanotheranostic agent for NIR-II imaging-guided synergetic photothermal/photodynamic therapy. Adv Ther. 2021;4(3):2000240.

[40] Yang S, Sun B, Liu F, Li N, Wang M, Wu P, Wu G-L, Fang H, He Y, Zhou W, et al. NIR-II imaging-guided mitochondrial-targeting organic nanoparticles for multimodal synergistic tumor therapy. Small. 2023;19(26):e2207995. 36942859 10.1002/smll.202207995

[41] Sun H, Zhang Q, Li J, Peng S, Wang X, Cai R. Near-infrared photoactivated nanomedicines for photothermal synergistic cancer therapy. Nano Today. 2021;37:Article 101073.

[42] Xiao H, Wang Y, Chen J, Xi S, Duan Z, Zhan Q, Tian Y, Wang L, Qu J, Liu R. NIR-II emissive superoxide radical photogenerator for photothermal/photodynamic therapy against hypoxic tumor. Adv Healthc Mater. 2023;13(20):Article e2303183. 10.1002/adhm.202303183 38117062

[43] Overchuk M, Weersink RA, Wilson BC, Zheng G. Photodynamic and photothermal therapies: Synergy opportunities for nanomedicine. ACS Nano. 2023;17(9):7979–8003. 37129253 10.1021/acsnano.3c00891 PMC10173698

[44] Sun H, Lv F, Liu L, Gu Q, Wang S. Conjugated polymer materials for photothermal therapy. Adv Ther. 2018;1(6):1800057.

[45] Tang Y, Bisoyi HK, Chen X-M, Liu Z, Chen X, Zhang S, Li Q. Pyroptosis-mediated synergistic photodynamic and photothermal immunotherapy enabled by a tumor-membrane-targeted photosensitive dimer. Adv Mater. 2023;35(25):Article e2300232. 36921347 10.1002/adma.202300232

[46] Cui D, Huang JG, Zhen X, Li JC, Jiang YY, Pu KY. A semiconducting polymer nano-prodrug for hypoxia-activated photodynamic cancer therapy. Angew Chem Int Ed Engl. 2019;58(18):5920–5924. 30793456 10.1002/anie.201814730

[47] Guo B, Huang Z, Shi Q, Middha E, Xu S, Li L, Wu M, Jiang J, Hu Q, Fu Z, et al. Organic small molecule based photothermal agents with molecular rotors for malignant breast cancer therapy. Adv Funct Mater. 2020;30(5):1907093.

[48] Jung HS, Verwilst P, Sharma A, Shin J, Sessler JL, Kim JS. Organic molecule-based photothermal agents: An expanding photothermal therapy universe. Chem Soc Rev. 2018;47(7):2280–2297. 29528360 10.1039/c7cs00522a PMC5882556

[49] Fan W, Huang P, Chen X. Overcoming the Achilles’ heel of photodynamic therapy. Chem Soc Rev. 2016;45(23):6488–6519. 27722560 10.1039/c6cs00616g

[50] Mou J, Lin T, Huang F, Chen H, Shi J. Black titania-based theranostic nanoplatform for single NIR laser induced dual-modal imaging-guided PTT/PDT. Biomaterials. 2016;84:13–24. 26803408 10.1016/j.biomaterials.2016.01.009

[51] Jin T, Cheng D, Jiang G, Xing W, Liu P, Wang B, Zhu W, Sun H, Sun Z, Xu Y, et al. Engineering naphthalimide-cyanine integrated near-infrared dye into ROS-responsive nanohybrids for tumor PDT/PTT/chemotherapy. Bioact Mater. 2022;14:42–51. 35310343 10.1016/j.bioactmat.2021.12.009 PMC8892148

[52] Chen Y, Lu Z, Wang D. Multifunctional nanoplatform for single NIR laser-regulated efficient PDT/PTT/chemotherapy. Biomacromolecules. 2024;25(2):1038–1046. 38242167 10.1021/acs.biomac.3c01100

[53] Wen K, Tan H, Peng Q, Chen H, Ma H, Wang L, Peng A, Shi Q, Cai X, Huang H. Achieving efficient NIR-II type-I photosensitizers for photodynamic/photothermal therapy upon regulating chalcogen elements. Adv Mater. 2022;34(7):Article e2108146. 34935224 10.1002/adma.202108146

[54] Duan X, Li J, Huang S, Li A, Zhang Y, Xue Y, Song X, Zhang Y, Hong S, Gao H, et al. Reusable and near-infrared light-activated zinc(II) metalated porphyrin with synergetic PDT/PTT for eradicating bacterial pneumonia. Chem Eng J. 2023;477:Article 146937.

[55] Wu D, Zhang Z, Li X, Zhou J, Cao Y, Qi S, Wang L, Liu Z, Yu G. Dynamically assembled nanomedicine based on host−guest molecular recognition for NIR laser-excited chemotherapy and phototheranostics. Acta Biomater. 2023;168:565–579. 37481192 10.1016/j.actbio.2023.07.022

[56] Gai S, Yang G, Yang P, He F, Lin J, Jin D, Xing B. Recent advances in functional nanomaterials for light–triggered cancer therapy. Nano Today. 2018;19:146–187.

[57] Gui Y, Wang Y, Wang D, Qin Y, Song G, Yan D, Tang BZ, Wang D. Thiophene π-bridge manipulation of NIR-II AIEgens for multimodal tumor phototheranostics. Angew Chem Int Ed Engl. 2024;63(14):Article e202318609. 38345594 10.1002/anie.202318609

[58] Lovell JF, Jin CS, Huynh E, Jin HL, Kim C, Rubinstein JL, Chan WCW, Cao WG, Wang LV, Zheng G. Porphysome nanovesicles generated by porphyrin bilayers for use as multimodal biophotonic contrast agents. Nat Mater. 2011;10(4):324–332. 21423187 10.1038/nmat2986

[59] Cai Y, Si W, Huang W, Chen P, Shao J, Dong X. Organic dye based nanoparticles for cancer phototheranostics. Small. 2018;14(25):Article e1704247. 29611290 10.1002/smll.201704247

[60] Jiang Y, Cui D, Fang Y, Zhen X, Upputuri PK, Pramanik M, Ding D, Pu K. Amphiphilic semiconducting polymer as multifunctional nanocarrier for fluorescence/photoacoustic imaging guided chemo-photothermal therapy. Biomaterials. 2017;145:168–177. 28866477 10.1016/j.biomaterials.2017.08.037

[61] Xu C, Pu K. Second near-infrared photothermal materials for combinational nanotheranostics. Chem Soc Rev. 2021;50(2):1111–1137. 33245316 10.1039/d0cs00664e

[62] Li J, Rao J, Pu K. Recent progress on semiconducting polymer nanoparticles for molecular imaging and cancer phototherapy. Biomaterials. 2018;155:217–235. 29190479 10.1016/j.biomaterials.2017.11.025 PMC5978728

[63] Ma J, Li P, Wang W, Wang S, Pan X, Zhang F, Li S, Liu S, Wang H, Gao G, et al. Biodegradable poly(amino acid)–gold–magnetic complex with efficient endocytosis for multimodal imaging-guided chemo-photothermal therapy. ACS Nano. 2018;12(9):9022–9032. 30059614 10.1021/acsnano.8b02750

[64] Li S, Deng Q, Li X, Huang Y, Li X, Liu F, Wang H, Qing W, Liu Z, Lee C-S. Bis-diketopyrrolopyrrole conjugated polymer nanoparticles as photothermic nanoagonist for specific and synergistic glioblastoma therapy. Biomaterials. 2019;216:Article 119252. 31212086 10.1016/j.biomaterials.2019.119252

[65] Li X, Liu L, Li S, Wan Y, Chen J-X, Tian S, Huang Z, Xiao Y-F, Cui X, Xiang C, et al. Biodegradable π-conjugated oligomer nanoparticles with high photothermal conversion efficiency for cancer theranostics. ACS Nano. 2019;13(11):12901–12911. 31682416 10.1021/acsnano.9b05383

[66] Hansen SF, Lennquist A. Carbon nanotubes added to the SIN list as a nanomaterial of very high concern. Nat Nanotechnol. 2020;15(1):3–4. 31925393 10.1038/s41565-019-0613-9

[67] Cheng L, Wang C, Feng L, Yang K, Liu Z. Functional nanomaterials for phototherapies of cancer. Chem Rev. 2014;114(21):10869–10939. 25260098 10.1021/cr400532z

[68] Zhao Z, Chen C, Wu W, Wang F, Du L, Zhang X, Xiong Y, He X, Cai Y, Kwok RTK, et al. Highly efficient photothermal nanoagent achieved by harvesting energy via excited-state intramolecular motion within nanoparticles. Nat Commun. 2019;10(1):768. 30770816 10.1038/s41467-019-08722-z PMC6377612

[69] Lyu Y, Zeng J, Jiang Y, Zhen X, Wang T, Qiu S, Lou X, Gao M, Pu K. Enhancing both biodegradability and efficacy of semiconducting polymer nanoparticles for photoacoustic imaging and photothermal therapy. ACS Nano. 2018;12(2):1801–1810. 29385336 10.1021/acsnano.7b08616

[70] Yin C, Li X, Wen G, Yang B, Zhang Y, Chen X, Zhao P, Li S, Li R, Wang L, et al. Organic semiconducting polymer amphiphile for near-infrared-II light-triggered phototheranostics. Biomaterials. 2020;232:Article 119684. 31901503 10.1016/j.biomaterials.2019.119684

[71] Antaris AL, Chen H, Cheng K, Sun Y, Hong G, Qu C, Diao S, Deng Z, Hu X, Zhang B, et al. A small-molecule dye for NIR-II imaging. Nat Mater. 2016;15(2):235–242. 26595119 10.1038/nmat4476

[72] Li X, Fang F, Sun B, Yin C, Tan J, Wan Y, Zhang J, Sun P, Fan Q, Wang P, et al. Near-infrared small molecule coupled with rigidness and flexibility for high-performance multimodal imaging-guided photodynamic and photothermal synergistic therapy. Nanoscale Horiz. 2021;6(2):177–185. 33443277 10.1039/d0nh00672f

[73] Zhang Z, Xu W, Kang M, Wen H, Guo H, Zhang P, Xi L, Li K, Wang L, Wang D, et al. An all-round athlete on the track of phototheranostics: Subtly regulating the balance between radiative and nonradiative decays for multimodal imaging-guided synergistic therapy. Adv Mater. 2020;32(36):Article e2003210. 32696561 10.1002/adma.202003210

[74] Geng J, Li K, Ding D, Zhang X, Qin W, Liu J, Tang BZ, Liu B. Lipid-PEG-folate encapsulated nanoparticles with aggregation induced emission characteristics: Cellular uptake mechanism and two-photon fluorescence imaging. Small. 2012;8(23):3655–3663. 22893564 10.1002/smll.201200814

[75] Xiao Y-F, Xiang C, Li S, Mao C, Chen H, Chen J-X, Tian S, Cui X, Wan Y, Huang Z, et al. Single-photomolecular nanotheranostics for synergetic near-infrared fluorescence and photoacoustic imaging-guided highly effective photothermal ablation. Small. 2020;16(34):Article e2002672. 32697430 10.1002/smll.202002672

[76] Antaris AL, Chen H, Diao S, Ma Z, Zhang Z, Zhu S, Wang J, Lozano AX, Fan Q, Chew L, et al. A high quantum yield molecule-protein complex fluorophore for near-infrared II imaging. Nat Commun. 2017;8:15269. 28524850 10.1038/ncomms15269 PMC5454457

[77] Sun Y, Qu C, Chen H, He M, Tang C, Shou K, Hong S, Yang M, Jiang Y, Ding B, et al. Novel benzo-bis(1,2,5-thiadiazole) fluorophores for in vivo NIR-II imaging of cancer. Chem Sci. 2016;7(9):6203–6207. 30034761 10.1039/c6sc01561a PMC6024204

[78] Zhang XD, Wang H, Antaris AL, Li L, Diao S, Ma R, Nguyen A, Hong G, Ma Z, Wang J, et al. Traumatic brain injury imaging in the second near-infrared window with a molecular fluorophore. Adv Mater. 2016;28(32):6872–6879. 27253071 10.1002/adma.201600706 PMC5293734

[79] Yang Q, Ma Z, Wang H, Zhou B, Zhu S, Zhong Y, Wang J, Wan H, Antaris A, Ma R, et al. Rational design of molecular fluorophores for biological imaging in the NIR-II window. Adv Mater. 2017;29(12):1605497. 10.1002/adma.201605497 28117499

[80] Yang Q, Hu Z, Zhu S, Ma R, Ma H, Ma Z, Wan H, Zhu T, Jiang Z, Liu W, et al. Donor engineering for NIR-II molecular fluorophores with enhanced fluorescent performance. J Am Chem Soc. 2018;140(5):1715–1724. 29337545 10.1021/jacs.7b10334

[81] Cai X, Liu X, Liao LD, Bandla A, Ling JM, Liu YH, Thakor N, Bazan GC, Liu B. Encapsulated conjugated oligomer nanoparticles for real-time photoacoustic sentinel lymph node imaging and targeted photothermal therapy. Small. 2016;12(35):4873–4880. 27439884 10.1002/smll.201600697

[82] Fan Q, Cheng K, Yang Z, Zhang R, Yang M, Hu X, Ma X, Bu L, Lu X, Xiong X, et al. Perylene-diimide-based nanoparticles as highly efficient photoacoustic agents for deep brain tumor imaging in living mice. Adv Mater. 2015;27(5):843–847. 25376906 10.1002/adma.201402972 PMC4347809

[83] Miao Q, Lyu Y, Ding D, Pu K. Semiconducting oligomer nanoparticles as an activatable photoacoustic probe with amplified brightness for in vivo imaging of pH. Adv Mater. 2016;28(19):3662–3668. 27000431 10.1002/adma.201505681

[84] Qi J, Li J, Liu R, Li Q, Zhang H, Lam JWY, Kwok RTK, Liu D, Ding D, Tang BZ. Boosting fluorescence-photoacoustic-Raman properties in one fluorophore for precise cancer surgery. Chem. 2019;5(10):2657–2677.

[85] Li S, Deng Q, Zhang Y, Li X, Wen G, Cui X, Wan Y, Huang Y, Chen J, Liu Z, et al. Rational design of conjugated small molecules for superior photothermal theranostics in the NIR-II biowindow. Adv Mater. 2020;32(33):Article e2001146. 32627868 10.1002/adma.202001146

[86] Shao W, Wei Q, Wang S, Li F, Wu J, Ren J, Cao F, Liao H, Gao J, Zhou M, et al. Molecular engineering of D-A-D conjugated small molecule nanoparticles for high performance NIR-II photothermal therapy. Mater Horiz. 2020;7:1379–1386.

[87] Xiao Y-F, Chen J-X, Li S, Tao W-W, Tian S, Wang K, Cui X, Huang Z, Zhang X-H, Lee C-S. Manipulating exciton dynamics of thermally activated delayed fluorescence materials for tuning two-photon nanotheranostics. Chem Sci. 2020;11(3):888–895. 10.1039/c9sc05817f PMC8145712 34123067

[88] Li L, Shao C, Liu T, Chao Z, Chen H, Xiao F, He H, Wei Z, Zhu Y, Wang H, et al. An NIR-II-emissive photosensitizer for hypoxia-tolerant photodynamic theranostics. Adv Mater. 2020;32(45):Article e2003471. 33029855 10.1002/adma.202003471

[89] He Z, Zhao L, Zhang Q, Chang M, Li C, Zhang H, Lu Y, Chen Y. An acceptor-donor-acceptor structured small molecule for effective NIR triggered dual phototherapy of cancer. Adv Funct Mater. 2020;30(16):1910301.

[90] Qi J, Fang Y, Kwok RTK, Zhang X, Hu X, Lam JWY, Ding D, Tang BZ. Highly stable organic small molecular nanoparticles as an advanced and biocompatible phototheranostic agent of tumor in living mice. ACS Nano. 2017;11(7):7177–7188. 28692799 10.1021/acsnano.7b03062

[91] Chen M, Zhang X, Liu J, Liu F, Zhang R, Wei P, Feng H, Tu M, Qin A, Lam JWY, et al. Evoking photothermy by capturing intramolecular bond stretching vibration-induced dark-state energy. ACS Nano. 2020;14(4):4265–4275. 32160460 10.1021/acsnano.9b09625

[92] Li X, Zhang D, Lu G, He T, Wan Y, Tse MK, Ren C, Wang P, Li S, Luo J, et al. Photochemical synthesis of nonplanar small molecules with ultrafast nonradiative decay for highly efficient phototheranostics. Adv Mater. 2021;33(38):Article e2102799. 34319622 10.1002/adma.202102799

[93] Li X, Zhang D, Yin C, Lu G, Wan Y, Huang Z, Tan J, Li S, Luo J, Lee C-S. A Diradicaloid small molecular nanotheranostic with strong near-infrared absorbance for effective cancer photoacoustic imaging and photothermal therapy. ACS Appl Mater Interfaces. 2021;13(14):15983–15991. 33788531 10.1021/acsami.0c21889

[94] Wan Y, Lu G, Wei WC, Huang YH, Li S, Chen JX, Cui X, Xiao YF, Li X, Liu Y, et al. Stable organic photosensitizer nanoparticles with absorption peak beyond 800 nanometers and high reactive oxygen species yield for multimodality phototheranostics. ACS Nano. 2020;14(8):9917–9928. 32706236 10.1021/acsnano.0c02767

[95] Cai Y, Liang P, Tang Q, Yang X, Si W, Huang W, Zhang Q, Dong X. Diketopyrrolopyrrole-triphenylamine organic nanoparticles as multifunctional reagents for photoacoustic imaging-guided photodynamic/photothermal synergistic tumor therapy. ACS Nano. 2017;11(1):1054–1063. 28033465 10.1021/acsnano.6b07927

[96] Wang Q, Xu J, Geng R, Cai J, Li J, Xie C, Tang W, Shen Q, Huang W, Fan Q. High performance one-for-all phototheranostics: NIR-II fluorescence imaging guided mitochondria-targeting phototherapy with a single-dose injection and 808 nm laser irradiation. Biomaterials. 2020;231:Article 119671. 31855624 10.1016/j.biomaterials.2019.119671

[97] Zhang R, Xu Y, Zhang Y, Kim HS, Sharma A, Gao J, Yang G, Kim JS, Sun Y. Rational design of a multifunctional molecular dye for dual-modal NIR-II/photoacoustic imaging and photothermal therapy. Chem Sci. 2019;10(36):8348–8353. 31803412 10.1039/c9sc03504d PMC6839587

[98] Wang Q, Xia B, Xu J, Niu X, Cai J, Shen Q, Wang W, Huang W, Fan Q. Biocompatible small organic molecule phototheranostics for NIR-II fluorescence/photoacoustic imaging and simultaneous photodynamic/photothermal combination therapy. Mater Chem Front. 2019;3:650–655.

[99] Chen C, Ou H, Liu R, Ding D. Regulating the photophysical property of organic/polymer optical agents for promoted cancer phototheranostics. Adv Mater. 2020;32(3):Article e1806331. 30924971 10.1002/adma.201806331

[100] Hong G, Antaris AL, Dai H. Near-infrared fluorophores for biomedical imaging. Nat Biomed Eng. 2017;1:0010.

[101] Jiang Z, Zhang C, Wang X, Yan M, Ling Z, Chen Y, Liu Z. A borondifluoride-complex-based photothermal agent with an 80 % photothermal conversion efficiency for photothermal therapy in the NIR-II window. Angew Chem Int Ed Engl. 2021;60(41):22376–22384. 34289230 10.1002/anie.202107836

[102] Zhang Y, Tao H, Li Q, Sheng W, Xu Y, Hao E, Chen M, Liu Z, Feng L. Surfactant-stripped J-aggregates of azaBODIPY derivatives: All-in-one phototheranostics in the second near infrared window. J Control Release. 2020;326:256–264. 32682904 10.1016/j.jconrel.2020.07.017

[103] Chen Y, Wang S, Zhang F. Near-infrared luminescence high-contrast in vivo biomedical imaging. Nat Rev Bioeng. 2023;1:60–78.

[104] Li J-B, Liu H-W, Fu T, Wang R, Zhang X-B, Tan W. Recent progress in small-molecule near-IR probes for bioimaging. Trends Chem. 2019;1(2):224–234. 32864595 10.1016/j.trechm.2019.03.002 PMC7453910

[105] Shen Q, Wang S, Yang N-D, Zhang C, Wu Q, Yu C. Recent development of small-molecule organic fluorophores for multifunctional bioimaging in the second near-infrared window. J Lumin. 2020;225:Article 117338.

[106] Miao Q, Pu K. Organic semiconducting agents for deep-tissue molecular imaging: Second near-infrared fluorescence, self-luminescence, and photoacoustics. Adv Mater. 2018;30(49):e1801778. 30058244 10.1002/adma.201801778

[107] He S, Song J, Qu J, Cheng Z. Crucial breakthrough of second near-infrared biological window fluorophores: Design and synthesis toward multimodal imaging and theranostics. Chem Soc Rev. 2018;47(12):4258–4278. 29725670 10.1039/c8cs00234g

[108] Cai Y, Wei Z, Song C, Tang C, Han W, Dong X. Optical nano-agents in the second near-infrared window for biomedical applications. Chem Soc Rev. 2019;48(1):22–37. 30444505 10.1039/c8cs00494c

[109] Jiang Y, Pu K. Advanced photoacoustic imaging applications of near-infrared absorbing organic nanoparticles. Small. 2017;13(30):1700710. 10.1002/smll.201700710 28597608

[110] Wang LV, Hu S. Photoacoustic tomography: In vivo imaging from organelles to organs. Science. 2012;335(6075):1458–1462. 22442475 10.1126/science.1216210 PMC3322413

[111] Guo Z, Zou Y, He H, Rao J, Ji S, Cui X, Ke H, Deng Y, Yang H, Chen C, et al. Bifunctional platinated nanoparticles for photoinduced tumor ablation. Adv Mater. 2016;28(46):10155–10164. 27714878 10.1002/adma.201602738

[112] He H, Ji S, He Y, Zhu A, Zou Y, Deng Y, Ke H, Yang H, Zhao Y, Guo Z, et al. Photoconversion-tunable fluorophore vesicles for wavelength-dependent photoinduced cancer therapy. Adv Mater. 2017;29(19):1606690. 10.1002/adma.201606690 28295684

[113] Yan D, Zhang Z, Zhang J, Li X, Wu Q, Gui Y, Zhu J, Kang M, Chen X, Tang BZ, et al. An all-rounder for NIR-II phototheranostics: Well-tailored 1064 nm-excitable molecule for photothermal combating of orthotopic breast cancer. Angew Chem Int Ed Engl. 2024;63(26):Article e202401877. 38637294 10.1002/anie.202401877

[114] Guo B, Sheng Z, Hu D, Liu C, Zheng H, Liu B. Through scalp and skull NIR-II photothermal therapy of deep orthotopic brain tumors with precise photoacoustic imaging guidance. Adv Mater. 2018;30(35):e1802591. 30129690 10.1002/adma.201802591

[115] Liu Y, Bhattarai P, Dai Z, Chen X. Photothermal therapy and photoacoustic imaging via nanotheranostics in fighting cancer. Chem Soc Rev. 2019;48(7):2053–2108. 30259015 10.1039/c8cs00618k PMC6437026

[116] Wen G, Li X, Zhang Y, Han X, Xu X, Liu C, Chan KWY, Lee C-S, Yin C, Bian L, et al. Effective phototheranostics of brain tumor assisted by near-infrared-II light-responsive semiconducting polymer nanoparticles. ACS Appl Mater Interfaces. 2020;12(30):33492–33499. 32627525 10.1021/acsami.0c08562

[117] Ge J, Jia Q, Liu W, Guo L, Liu Q, Lan M, Zhang H, Meng X, Wang P. Red-emissive carbon dots for fluorescent, photoacoustic, and thermal theranostics in living mice. Adv Mater. 2015;27(28):4169–4177. 26045099 10.1002/adma.201500323

[118] Lee M-Y, Lee C, Jung HS, Jeon M, Kim KS, Yun SH, Kim C, Hahn SK. Biodegradable photonic melanoidin for theranostic applications. ACS Nano. 2016;10(1):822–831. 26623481 10.1021/acsnano.5b05931

[119] Kim C, Favazza C, Wang LV. In vivo photoacoustic tomography of chemicals: High-resolution functional and molecular optical imaging at new depths. Chem Rev. 2010;110(5):2756–2782. 20210338 10.1021/cr900266s PMC2872199

[120] Smith BR, Gambhir SS. Nanomaterials for in vivo imaging. Chem Rev. 2017;117(3):901–986. 28045253 10.1021/acs.chemrev.6b00073

[121] Chen H, Zhang J, Chang K, Men X, Fang X, Zhou L, Li D, Gao D, Yin S, Zhang X, et al. Highly absorbing multispectral near-infrared polymer nanoparticles from one conjugated backbone for photoacoustic imaging and photothermal therapy. Biomaterials. 2017;144:42–52. 28822291 10.1016/j.biomaterials.2017.08.007

[122] Zhang X, Lin S, Zhao F, Zhang J, Lei S, Bai F, Liu Q, Wu J, He T, Huang P, et al. Programmably controllable delivery of metastable ferrous ions for multiscale dynamic imaging guided photothermal primed chemodynamic therapy. Adv Mater. 2023;35(25):Article e2210876. 36870077 10.1002/adma.202210876

[123] Gao X, Jiang S, Li C, Chen Y, Zhang Y, Huang P, Lin J. Highly photostable croconium dye-anchored cell membrane vesicle for tumor pH-responsive duplex imaging-guided photothermal therapy. Biomaterials. 2021;267:Article 120454. 33160122 10.1016/j.biomaterials.2020.120454

[124] Lei S, Zhang Y, Blum NT, Huang P, Lin J. Recent advances in croconaine dyes for bioimaging and theranostics. Bioconjugate Chem. 2020;31(9):2072–2084. 10.1021/acs.bioconjchem.0c00356 32786372

[125] Wang J, Li Y, Nie G. Multifunctional biomolecule nanostructures for cancer therapy. Nat Rev Mater. 2021;6:766–783. 34026278 10.1038/s41578-021-00315-x PMC8132739

[126] Yang Z, Chen X. Semiconducting perylene diimide nanostructure: Multifunctional phototheranostic nanoplatform. Acc Chem Res. 2019;52(5):1245–1254. 30977625 10.1021/acs.accounts.9b00064

[127] Thangudu S, Kaur N, Korupalli C, Sharma V, Kalluru P, Vankayala R. Recent advances in near infrared light responsive multi-functional nanostructures for phototheranostic applications. Biomater Sci. 2021;9(16):5472–5483. 34269365 10.1039/d1bm00631b

[128] Fan W, Yung B, Huang P, Chen X. Nanotechnology for multimodal synergistic cancer therapy. Chem Rev. 2017;117(22):13566–13638. 29048884 10.1021/acs.chemrev.7b00258

[129] Ding F, Feng J, Zhang X, Sun J, Fan C, Ge Z. Responsive optical probes for deep-tissue imaging: Photoacoustics and second near-infrared fluorescence. Adv Drug Deliv Rev. 2021;173:141–163. 33774116 10.1016/j.addr.2021.03.008

[130] Speed CA. Therapeutic ultrasound in soft tissue lesions. Rheumatology. 2001;40(12):1331–1336. 11752501 10.1093/rheumatology/40.12.1331

[131] Sakdinawat A, Attwood D. Nanoscale X-ray imaging. Nat Photonics. 2010;4:840–848.

[132] Huang J, Su L, Xu C, Ge X, Zhang R, Song J, Pu K. Molecular radio afterglow probes for cancer radiodynamic theranostics. Nat Mater. 2023;22(11):1421–1429. 37667071 10.1038/s41563-023-01659-1

[133] Wang X, Sun W, Shi H, Ma H, Niu G, Li Y, Zhi J, Yao X, Song Z, Chen L, et al. Organic phosphorescent nanoscintillator for low-dose X-ray-induced photodynamic therapy. Nat Commun. 2022;13:5091. 36042210 10.1038/s41467-022-32054-0 PMC9428140

